# Enriching productive mutational paths accelerates enzyme evolution

**DOI:** 10.1038/s41589-024-01712-3

**Published:** 2024-09-11

**Authors:** David Patsch, Thomas Schwander, Moritz Voss, Daniela Schaub, Sean Hüppi, Michael Eichenberger, Peter Stockinger, Lisa Schelbert, Sandro Giger, Francesca Peccati, Gonzalo Jiménez-Osés, Mojmír Mutný, Andreas Krause, Uwe T. Bornscheuer, Donald Hilvert, Rebecca M. Buller

**Affiliations:** 1 Competence Center for Biocatalysis, Zurich University of Applied Sciences, Waedenswil, Switzerland; 2 Department of Biotechnology and Enzyme Catalysis, University of Greifswald, Greifswald, Germany; 3 Center for Functional Protein Assemblies & Department of Bioscience, TUM School of Natural Sciences, Technical University of Munich (TUM), Garching, Germany; 4 Department of Biotechnology, Delft University of Technology, Delft, The Netherlands; 5 Center for Cooperative Research in Biosciences (CIC bioGUNE), Basque Research and Technology Alliance (BRTA), Derio, Spain; 6 Ikerbasque, Basque Foundation for Science, Bilbao, Spain; 7 Department of Computer Science, ETH Zurich, Zurich, Switzerland; 8 Laboratory of Organic Chemistry, ETH Zurich, Zurich, Switzerland

**Keywords:** High-throughput screening, X-ray crystallography, Biocatalysis, Enzyme mechanisms

## Abstract

Darwinian evolution has given rise to all the enzymes that enable life on Earth. Mimicking natural selection, scientists have learned to tailor these biocatalysts through recursive cycles of mutation, selection and amplification, often relying on screening large protein libraries to productively modulate the complex interplay between protein structure, dynamics and function. Here we show that by removing destabilizing mutations at the library design stage and taking advantage of recent advances in gene synthesis, we can accelerate the evolution of a computationally designed enzyme. In only five rounds of evolution, we generated a Kemp eliminase—an enzymatic model system for proton transfer from carbon—that accelerates the proton abstraction step >10^8^-fold over the uncatalyzed reaction. Recombining the resulting variant with a previously evolved Kemp eliminase HG3.17, which exhibits similar activity but differs by 29 substitutions, allowed us to chart the topography of the designer enzyme’s fitness landscape, highlighting that a given protein scaffold can accommodate several, equally viable solutions to a specific catalytic problem.

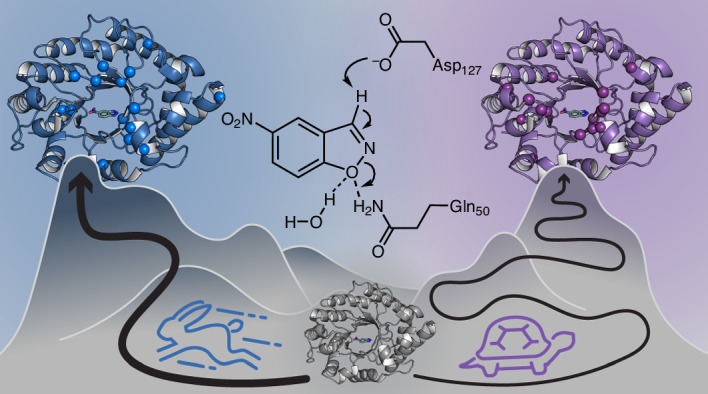

## Abstract (web summary)

By enriching productive mutational paths, a Kemp eliminase that speeds up proton abstraction >10^8^-fold was developed in only five evolution rounds. Recombining it with a variant differing by 29 substitutions revealed the underlying fitness landscape.

## Main

Natural evolution routinely generates powerful biocatalysts by elegantly navigating protein sequence space^1^. John Maynard Smith likened this process to a journey between functional proteins in a vast landscape^2^. Active enzymes, although rare, are connected to other functional catalysts in a continuous network that can be traversed by unit mutational steps. This connectivity is crucial for successful evolution via point mutations; without it, single mutations would be highly likely to reduce fitness, halting evolution^2^. Protein engineers have learned to traverse this sequence network through ‘directed evolution’, which involves iterative cycles of mutagenesis and screening or selection to develop improved proteins in the laboratory^3^. This deliberate exploration of sequence space revealed that proteins can rapidly adapt to new selection criteria, implying ‘easily’ navigable paths within the fitness landscape^3–6^. Following these paths has led to the improvement of diverse proteins for industrial or therapeutic applications^7^.

Nevertheless, a definitive understanding of the structural and molecular factors that govern the efficiency with which an enzyme catalyzes a reaction has remained elusive^3^, preventing scientists from reliably predicting amino acid sequences that encode a specific function. Today, enzyme engineering still relies on testing large libraries of variants, consisting of hundreds to millions of distinct protein sequences, to identify solutions suitable for application^8^. Even considering advanced high-throughput screening and computational methods^7^, the laboratory evolution of enzymes remains a resource- and time-consuming process^9,10^. Unfortunately, identifying the shortest evolutionary path to attain a desired protein function remains challenging as many evolutionary trajectories lead downhill, away from improved function. Most single-site mutations are either deleterious (30–50%) or neutral (50–70%), with only as few as 1% of residue exchanges being beneficial^3,11–15^. To increase the efficiency of the search process, limiting sequence space exploration to favorable amino acid exchanges would consequently be beneficial. While predicting which mutations will improve function and are thus worth exploring is a difficult task^16–18^, the prediction and exclusion of deleterious mutations is a potentially much more straightforward exercise^19^.

**Fig. 1:**
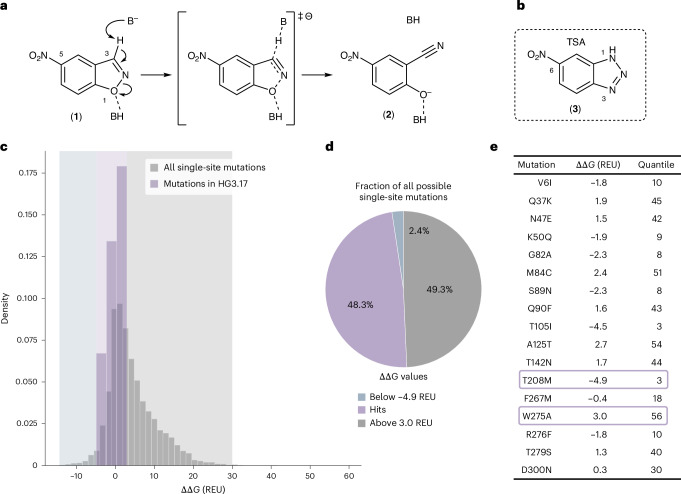
Stability predictions guide library design of Kemp eliminase HG3. **a**, The Kemp elimination reaction proceeds through deprotonation of 5-nitrobenzisoxazole (**1**) to afford salicylonitrile (**2**). The double dagger indicates the transition state of the reaction. **b**, Structure of the TSA 6-nitrobenzotriazole (**3**). **c**, Density plot of predicted ΔΔ*G* values for HG3 sequences containing single-point mutations (lower values correspond to higher predicted stability). The gray histogram depicts the ΔΔ*G* values of all possible single-site HG3 variants (5,758 variants including wild type, covering residues E1 to Q303). The purple histogram depicts the distribution of the ΔΔ*G* values for HG3 variants, each containing a single beneficial mutation from HG3.17. **d**, In total, 48.3% (2,781) of all possible single-site HG3 variants (purple-shaded area) exhibit predicted ΔΔ*G* values that lie within the ΔΔ*G* interval defined by the single-site variants containing the most and the least stabilizing mutation found in HG3.17 (HG3 T208M, −4.9 REU and HG3 W275A, 3 REU). Comparatively, 2.4% (138) of all HG3 single-site variants are predicted to contain a more stabilizing mutation (blue-shaded area) and 49.3% (2,839) of all single-site variants are predicted to contain a more destabilizing mutation (gray-shaded area). **e**, List of ΔΔ*G* values (given in REU) for HG3 sequences containing beneficial single mutations from HG3.17. The most (T208M) and least (W275A) stabilizing mutations are highlighted with a purple box. Source data

Here we demonstrate that removing deleterious mutations from protein libraries can greatly accelerate the search of protein fitness landscapes. For this purpose, we selected the de novo-designed Kemp eliminase HG3 as a test system^20,21^. HG3 catalyzes the Kemp elimination, a model for proton transfer from carbon (Fig. 1a)^22^. It was generated using quantum mechanical (QM) calculations to predict an idealized active site pocket for transition state stabilization^20,23^. Although the artificial enzyme showed modest activity compared to other de novo designs and catalytic antibodies elicited for the same reaction^24,25^, it was optimized over 17 rounds of mutagenesis and screening^21^. The improved variant HG3.17, which contained 17 additional mutations, was more active, better expressed and more thermostable compared to its ancestor^21^ and approaches the efficiency of natural enzymes that promote metabolically important proton transfers^21,26^. A retrospective analysis based on X-ray crystallography and computation showed that eight of HG3.17’s mutations are sufficient to reach ~80% of its activity^27^.

## Results

### Enzyme library design and construction

For rapid optimization of Kemp eliminase HG3, we designed complex gene libraries that excluded destabilizing mutations. The latter were identified by calculating ΔΔ*G* values for the free energy change upon mutation^28^ for all 5,757 possible single amino acid substitutions in the HG3 sequence (19 (non-wild-type amino acids) × 303 (sequence length)) using a cartesian ΔΔ*G* protocol implemented in the Rosetta Protein Modeling Suite^29^. Notably, this analysis indicated that approximately half of all possible single-site mutations (49.3%; 2,839 of 5,758) in HG3.17 could have been removed from the library design space without losing a single successful mutation (Fig. 1c–e).

**Fig. 2:**
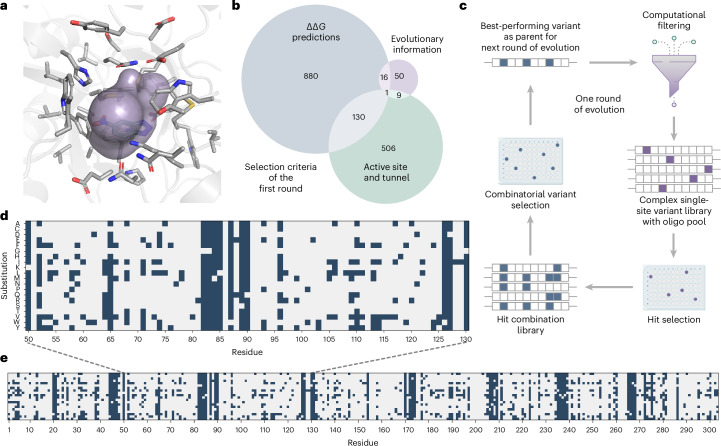
Library design and variant selection strategy. **a**, All HG3 residues (shown in gray sticks) within a 6 Å radius around the TSA 6-nitrobenzotriazole (**3**) as well as residues lining the active site entry tunnel (purple area, identified with Caver 3.0 (ref. ^49^)) were selected as ‘active site and tunnel residues’ and fully saturated. For each round, the TSA (**3**) was placed by aligning the crystal structure of HG3.17 (PDB 4BS0) to an AlphaFold^50^ model of the round’s parent (depicted here: HG3). **b**, HG3 libraries were designed by including all single-site variants with a predicted ΔΔ*G* below −0.5 REU, all variants obtainable by fully saturating the active site and tunnel residues as well as single-site variants derived from a HotSpot Wizard^30^ analysis. **c**, Schematic representation of a full cycle of engineering, consisting of computational filtering, constructing and screening of the complex single-site variant libraries to identify improved variants, followed by analyzing a combinatorial library built from identified beneficial mutations (hit combination library). The best-performing variant identified in the hit combination library served as the parent for the next round of engineering. **d**, Heatmap representation of the complex single-site variant library of the first round, highlighting the substitution pattern between sites 50 and 130. Amino acid substitutions, which are included in the oligo pools, are indicated as blue squares (for example, residue 50 is fully saturated excluding the wild-type amino acid lysine), **e**, Heatmap representation of the entire library design underscoring the attainable library complexity using oligo pools. Source data

Using this finding to guide library design, we fully saturated residues found within a 6 Å radius of the bound 6-nitrobenzotriazole (**3**) as well as all residues lining the tunnel leading to the active site (Fig. 2a) to avoid potential bias caused by knowledge from prior evolution experiments^21^. Additional mutations were only considered if the predicted ΔΔ*G* of the resulting variant was below −0.5 Rosetta Energy Units (REU). This energy threshold was set for experimental reasons (oligo pool size and screening capacity) and limited the screening effort to 30% of all possible single-site mutations (ca. 1,800 variants per round). In addition, a small number of single-site mutations suggested by a HotSpot Wizard^30^ analysis, which is based on sequence, structure and evolutionary information, were included (Fig. 2b).

**Extended Data Fig. 1:**
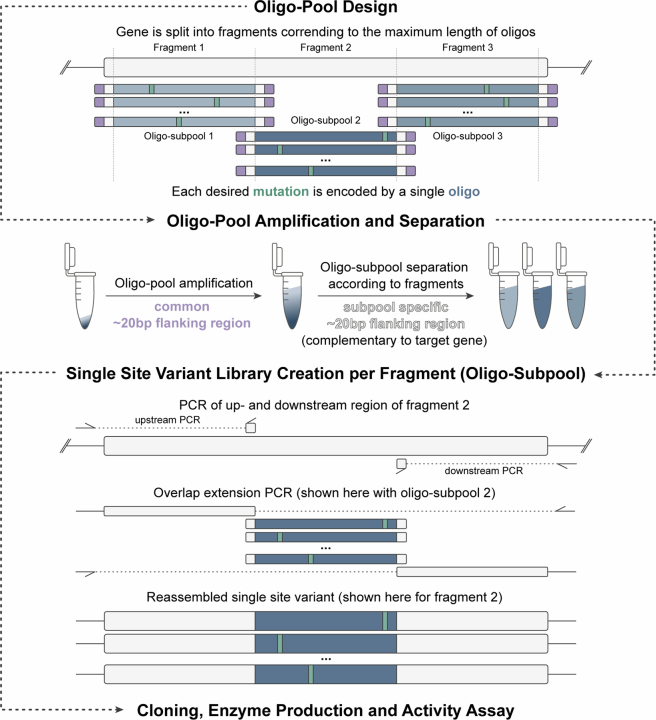
Creation of complex single-site variant libraries using oligo pools. The gene is split into an appropriate number of fragments corresponding to the maximum length of the available oligos (200 bp in our case). For each desired single-site variant, an individual oligo is designed and later used in an overlap extension PCR with appropriate flanking regions, in this way introducing the desired mutations. In each oligo, a common flanking region (purple) is included, allowing the oligo’s initial amplification from the low-concentrated oligo pool. Another flanking region complementary to the gene (white) permits to specifically amplify oligos covering a certain gene fragment (subpool-specific amplification). Each variant gene is reassembled using an overlap extension PCR using the amplified oligo subpool in combination with gene fragments generated from appropriate upstream and downstream PCRs.

**Extended Data Fig. 2:**
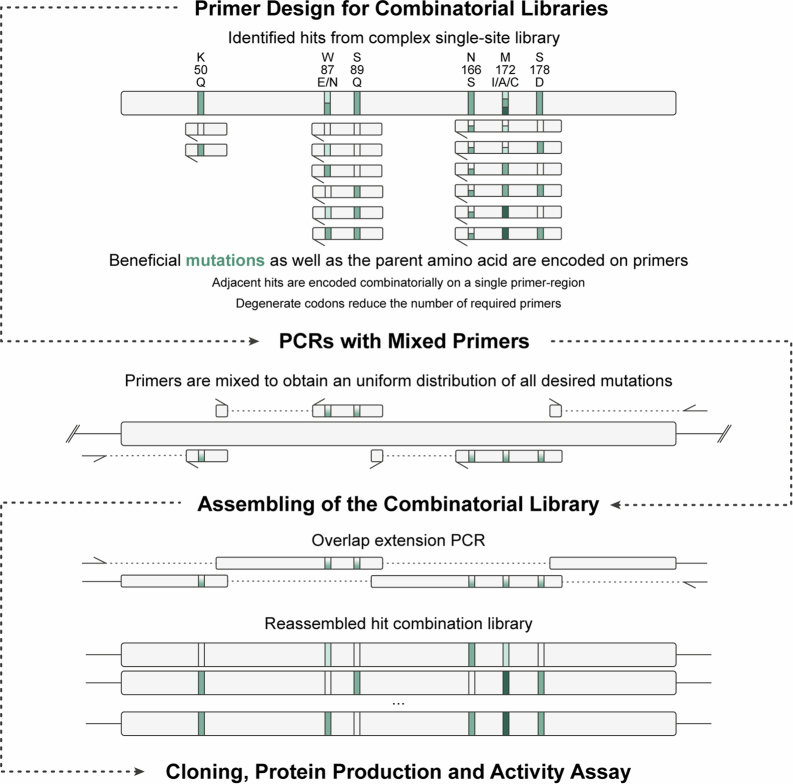
Creation of combinatorial hit libraries. Identified beneficial mutations are encoded on customized primers. If appropriate, adjacent mutations are grouped on one primer, and degenerate codons are used to cover several amino acids at the same site reducing the overall number of required primers. Next, the gene is amplified in fragments spanning regions between mutations. Finally, an overlap extension PCR is performed to reassemble all mutagenized fragments into the final variant library.

The single-site variant libraries were physically constructed using mixtures of unique DNA oligonucleotides (oligo pools) of limited length (200 bp) covering the entire HG3 gene. Full genes were assembled by overlap extension PCR^31^ using eight customized oligo fragments (Fig. 2d,e and Extended Data Fig. 1). The resulting gene libraries were used to transform *Escherichia coli* BL21 (DE3), and the corresponding Kemp eliminase variants were produced and assayed in cell lysates by following the formation of colored salicylonitrile (**2**) at 380 nm. Beneficial single mutations were combined in small combinatorial libraries and screened to identify the parent for the next evolution cycle (Extended Data Fig. 2).

**Fig. 3:**
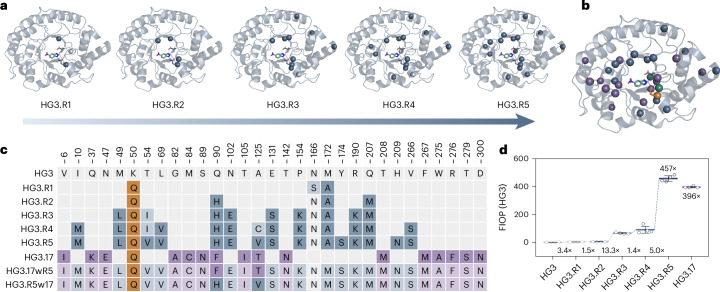
Evolutionary trajectory of HG3.R5. **a**, The evolutionary intermediates from each optimization round are depicted, showing the acquired mutations as dark blue spheres. The light blue spheres indicate transient mutations that undergo further substitution during evolution. **b**, Comparison of residues mutated in HG3.R5 (blue spheres) and HG3.17 (purple spheres), highlighting the two shared sites Q90 and A125 (green spheres) and the common mutation K50Q (orange sphere). The catalytic dyad (D127 and Q50) is shown in stick representation. **c**, Overview of the mutations acquired over the five rounds of evolution to yield HG3.R5, allowing a comparison to the mutations present in HG3.17. Mutation patterns for HG3.17 and the combinatorial variants HG3.R5w17 and HG3.17wR5 are included for comparison. **d**, Graph highlighting the FIOP of the intermediate and final Kemp variants for the cleavage of 5-nitrobenzisoxazole (**1**) under plate screening conditions. The data are obtained from four independent replicates and presented as mean ± s.d. The blue dashed line connects variants from the HG3.R5 trajectory. The FIOP of HG3.17 assayed under the same conditions is depicted for comparison. Structural illustrations are adapted from PDB 8RD5. FIOP, fold improvement over parent. Source data

This engineering procedure was carried out five times in total. Each cycle consisted of computational filtering (stability predictions in the frame of the parent gene), constructing and screening of the single-site variant libraries to identify favorable amino acid substitutions (on average, five to ten beneficial substitutions per round were identified showing a 1.2- to 2.4-fold improvement over the parent enzyme), followed by analyzing a combinatorial library built from identified beneficial mutations. Using this strategy, Kemp eliminase activity was boosted by a factor of >450-fold (Figs. 2c and 3d and Extended Data Fig. 2). Because oligos synthesized as pools can contain errors^32–34^, we sequenced the initial libraries and confirmed a coverage of >50% of the targeted mutations. We judged this acceptable as our enzyme engineering protocol was designed to interrogate all nondestabilizing mutations in each engineering cycle. In analogy to natural evolution, this sampling strategy allowed us to observe instances of unit mutational step reversals (round 1: N166S/round 2: S166N) and the stepwise finetuning of key amino acid positions (round 3: T54I/round 5: I54V and round 4: A125C/round 5: C125V; Fig. 3a,c).

### Biochemical characterization of optimized Kemp eliminases

**Extended Data Fig. 3:**
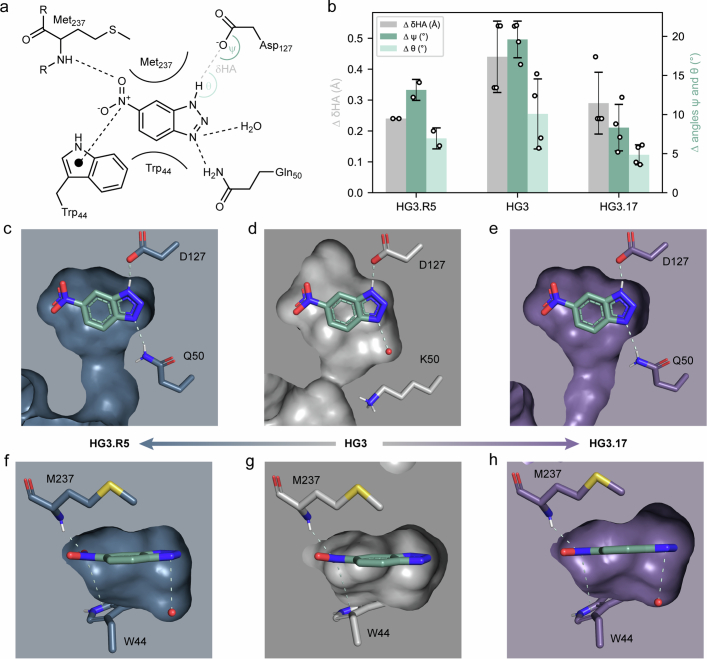
Active site architecture of HG3 variants. **a**, Schematic representation of ligand–enzyme interactions found in HG3.R5. The TSA (**3**) interacts via hydrogen bonds with the backbone amide of M237 as well as the side chains of Q50 (oxyanion stabilization) and D127 (catalytic base). Additionally, the nitro group of the ligand is anchored by van der Waals interactions to the side chain of W44. Of note, the TSA (**3**) forms a hydrogen bond to an evolutionarily acquired water in the active site of HG3.R5. **b**, Evolutionary optimization of the angles and distances characterizing the hydrogen-bonding interaction between D127 and the TSA (**3**). Values are given as the difference (∆) between the optimal angles and distances calculated for hydrogen-bonding interactions between acetamide dimers (δHA = 1.94 Å; θ = 159.4°; ψ = 112.3°)^77^ and the measured values from the binary crystal structures. In detail, measured distances and angles were obtained from all unique assemblies in the asymmetric unit cell of HG3.R5 (2 assemblies, 8RD5), HG3 (4 assemblies, 5RGA and 7K4Q) and HG3.17 (4 assemblies from 5RGE, 7K4Z and 4BS0). The data are obtained from the above-mentioned assemblies using Pymol (2.5.5) and presented as mean ± SD. **c**–**h**, Cut-away view of the TSA (**3**) bound active site of HG3.R5 (blue, **c** and **f**), HG3 (gray, **d** and **g**) and HG3.17 (purple, **e** and **h**), highlighting the improved shape complementarity of active site and ligand in the evolved variants. Structural illustrations are adapted from the PDB (HG3: 5RGA, HG3.R5: 8RD5, HG3.17: 5RGE). Source data

Thanks to this filtering strategy, only five rounds of evolution were needed to identify the highly active Kemp eliminase HG3.R5. Notably, HG3.R5 is characterized by 16 mutations, only three of which target the same residues (K50, Q90 and A125) as HG3.17. Overall, however, the two proteins have only one mutation in common, K50Q, which stabilizes the developing negative charge in the transition state (Extended Data Fig. 3)^21,27^. Although a comparison of the HG3.17 and HG3.R5 sequences suggests that the choice and placement of key catalytic groups exhibit little flexibility (the same catalytic dyad consisting of D127 and K50Q appeared independently in both cases), the rest of the active site displays considerable freedom with respect to the type and positioning of amino acid substitutions (Fig. 3b,c).

To assess the catalytic improvements gained during evolution, we determined the steady-state enzyme turnover number *k*_cat_ and Michaelis constant *K*_m_ parameters for the cleavage of 5-nitrobenzisoxazole (**1**) of the best variants from each evolution round by numerically fitting the total time course data (Supplementary Figs. 1 and 2). Although we corrected the data for variants HG3 and HG3.17 for conformational selection^35^, we assumed that the Kemp variants of the HG3.R series were fully active as isolated, potentially underestimating the true efficiency of these catalysts. This analysis showed that HG3.R5 cleaves 5-nitrobenzisoxazole (**1**) with a *k*_cat_ of 702 ± 79 s^−1^ (mean ± s.d.) and a *k*_cat_/*K*_m_ of 1.7 × 10^5^ M^−1^ s^−1^. The >200-fold improvement in catalytic efficiency over the original computational design (Table 1) is comparable to that achieved by HG3.17 and can be largely attributed to an increase in turnover number rather than substrate affinity (Table 1).

**Table 1:** Steady-state parameters and melting temperatures of representative Kemp eliminases

Variant	*k*_cat_ (s^−1^)^a^	*K*_m_ (mM)^a^	*k*_cat_/*K*_m_ (M^−1^ s^−1^)^b^	Melting temp. (°C)
HG3^c^	6.5 ± 2.3	9.7 ± 3.9	4.1 x 10^2^ ± 0.3 x 10^2^	53.7 ± 0.1
HG3.R1^e^	48 ± 25	25 ± 14	1.9 x 10^3^ ± 0.4 x 10^3^	59.0 ± 0.1
HG3.R2^e^	110 ± 35	22 ± 6	4.3 x 10^3^ ± 1.0 x 10^3^	65.2 ± 0.1
HG3.R3^e^	143 ± 52	8.8 ± 3.4	1.2 x 10^4^ ± 0.2 x 10^4^	61.0 ± 0.1
HG3.R4^e^	561 ± 90	6.7 ± 1.2	6.7 x 10^4^ ± 0.5 x 10^4^	59.4 ± 0.1
HG3.R5^e^	702 ± 79	4.8 ± 0.6	1.7 x 10^5^ ± 0.2 x 10^5^	60.5 ± 0.1
HG3.17^d^	604 ± 30	3.5 ± 0.2	1.5 x 10^5^ ± 0.3 x 10^5^	56.5 ± 0.1
HG3.17wR5^e^	74 ± 45	20 ± 13	3.1 x 10^3^ ± 0.1 x 10^3^	56.2 ± 0.1
HG3.R5w17^e^	19.3 ± 6.7	7.8 ± 3.0	4.3 x 10^3^ ± 0.1 x 10^3^	64.2 ± 0.1

Values are mean ± s.d. of three independent measurements.

^a^Steady-state parameters *k*_cat_ and *K*_m_ were derived by numerically fitting the total time course data for the cleavage of 5-nitrobenzisoxalzole (**1**).

^b^Catalytic efficiencies (*k*_cat_/*K*_m_) were calculated from the slope of the linear portion ([S] ≪ *K*_m_) of the Michaelis–Menten model.

^c^*k*_2_ = 2.32 x 10^−4^ s^−1^ and *k*_−2_ = 8.12 x 10^−5^ s^−1^.

^d^*k*_2_ = 16.7 x 10^−4^ s^−1^ and *k*_−2_= 6.68 x 10^−5^ s^−1^.

^e^Enzyme was assumed to be fully present in the active state.

### TSA bound structure of Kemp eliminase HG3.R5

**Fig. 4:**
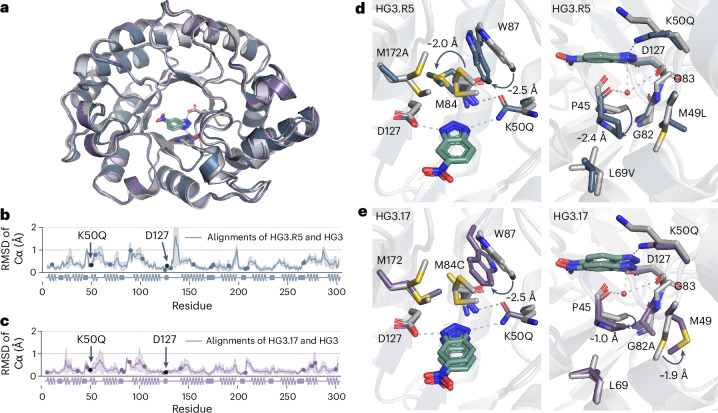
Structural comparison of HG3, HG3.17 and HG3.R5. **a**, Overlay of the cartoon representation of HG3.R5 (blue, PDB 8RD5), HG3 (gray, PDB 5RGA) and HG3.17 (purple, PDB 5RGE) with bound TSA (**3**). **b**, Root mean square deviation (RMSD) (Cα) between HG3 and HG3.R5 with bound ligand (**3**) (*n* = 8 pairwise alignments of the biological assemblies in the asymmetric unit of PDB 5RGA, 7K4Q and 8RD5). Blue spheres represent mutations in HG3.R5. **c**, RMSD (Cα) between HG3 and HG3.17 with bound TSA (**3**) (*n* = 16 pairwise alignments of the biological assemblies in the asymmetric unit of PDB 5RGA, 5RGE, 7K4Q, 7K4Z and 4BS0). Purple spheres highlight mutations in HG3.17, while black spheres denote catalytic residues K50Q and D127 (**b**,**c**). The uncertainty of the pairwise alignments is represented by shading (**b**,**c**). **d**, Overlay of the active sites of HG3 (light gray) and HG3.R5 (blue) highlights the effect of mutation M172A (left) on the conformation of M84 (movement of ∼2 Å) and W87 (movement of ∼2.5 Å) as well as of mutations M49L and L69V (right) on the conformation of P45 (movement of ∼2.4 Å) providing space for the binding of a water molecule (red sphere) not present in the original design HG3. **e**, Overlay of the active sites of HG3 (light gray) and HG3.17 (purple) highlights that a similar side chain movement of W87 (∼2.5 Å) is enabled by mutation M84C (left), while a conformational change of P45 (∼1 Å) and M49 (∼1.9 Å) is likely triggered through the proximate mutation G82A (right). The latter conformational changes allow the binding of a water molecule (red sphere) not present in the original design HG3. Pymol (2.5.5) was used to measure distances and present structures. Source data

Notably, while the evolutionary trajectories of HG3.R5 and HG3.17 diverged at the nucleotide and amino acid level, we found that similar structural effects drive catalysis in both enzymes. Alignment of the 1.5 Å X-ray structure of HG3.R5 (Protein Data Bank (PDB) 8RD5; Supplementary Table 1) in complex with 6-nitrobenzotriazole (**3**) with HG3 and HG3.17 (Fig. 4a–c) showed the deeply buried active site that characterizes all variants of the HG3 series and shields the ligand from bulk solvent. Like HG3.17, decisive structural elements in HG3.R5 include the K50Q mutation (oxyanion stabilization) as well as excellent shape complementarity to the transition state analog (**3**) (TSA (**3**); Extended Data Fig. 3c,f), including fine-tuned interactions between the catalytic base, D127, and the acidic N–H bond of 6-nitrobenzotriazole (**3**) (Extended Data Fig. 3b).

**Extended Data Fig. 4:**
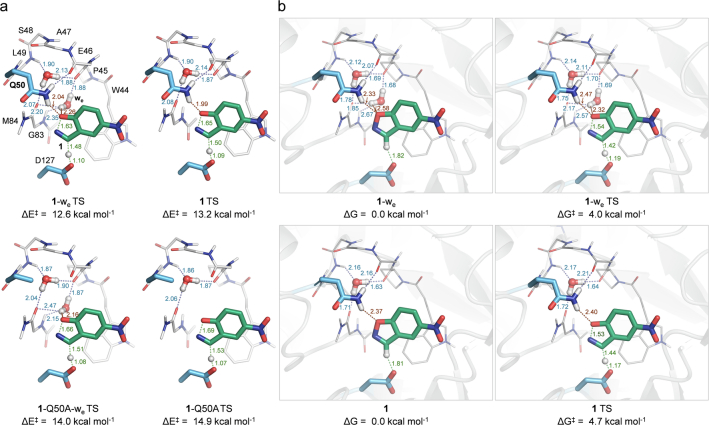
Quantum mechanical calculations of the Kemp elimination catalyzed by HG3.R5. **a**, Cluster model transition structures (full QM). **b**, Enzyme–substrate complex and transition structures (hybrid QM/MM). The TS stabilization exerted by crystallographic water *w*_*e*_ was calculated to be similar and additive to that achieved by the side chain of Q50. Note the shorter and better-oriented polar contact between the substrate and the water molecule in the TS, reflecting its contribution to oxyanion stabilization. A high degree of preorganization for catalysis was found in the X-ray structures and is reflected in the very low computed reaction barriers. The cluster QM calculated activation energies are incidentally in good agreement with the experimentally measured turnover number (k_cat_ = 702 s^−1^; ΔG^‡^ ≈ 13.6 kcal mol^−1^ at 25 °C), while the QM/MM ones are substantially lower despite using the same DFT functional and a similar basis set. In this regard, a dramatic influence of the level of theory and implicit solvation on the calculated and ΔE^‡^ and ΔG^‡^ was found. Hence, attention should be focused on relative rather than absolute activation barriers. Pymol (2.4.0a) was used for structural representations.

The binding of an ordered water molecule in a position to stabilize the developing negative charge in the transition state is also notable. In HG3.R5, a substantial movement of P45 away from the ligand (2.4 Å) provided space for the water molecule (*w*_*e*_), which is embedded in a dense hydrogen-bonding network and forms a polar contact with N3 (3.4 Å) of the TSA (**3**) (Fig. 4d and Extended Data Fig. 3f). A similarly placed water is also found in the structure of HG3.17 (ref. ^36^; Fig. 4e and Extended Data Fig. 3h), but it is accommodated by a different set of mutations (Fig. 4e). QM calculations on a 5-nitrobenzisoxazole (**1**) bound cluster model derived from the binary X-ray structure of HG3.R5 (PDB 8RD5) suggest that water *w*_*e*_ is a weak oxyanion binder that stabilizes the transition state by 0.6 kcal mol^−1^ (Extended Data Fig. 4a), a value which is confirmed by hybrid quantum mechanics/molecular mechanics (QM/MM) full optimization of the Michaelis–Menten complex and the transition state bound at the active site of the fully solvated enzyme (Extended Data Fig. 4b). Interestingly, this stabilizing effect is similar to that exerted by the Q50 side chain (1.4 kcal mol^−1^), and the combined effect of these interactions amounts to 2.3 kcal mol^−1^ (Extended Data Fig. 4a), suggesting some degree of cooperativity. In analogy to the autonomous evolution of oxyanion holes in natural enzymes, in which water molecules are often found to contribute critical hydrogen bonds^37^, this catalytic element arose independently in the evolution trajectories of both HG series, indicating water’s usefulness in the Kemp elimination reactions. Of note, initial in silico designs of Kemp eliminases included water as an alternative to amino acids as hydrogen bond donors^23^ due to its flexibility and ability to solvate the developing negative charge in the transition state^38^.

### HG3.R5 substitutions form an intricate interaction network

**Extended Data Fig. 5:**
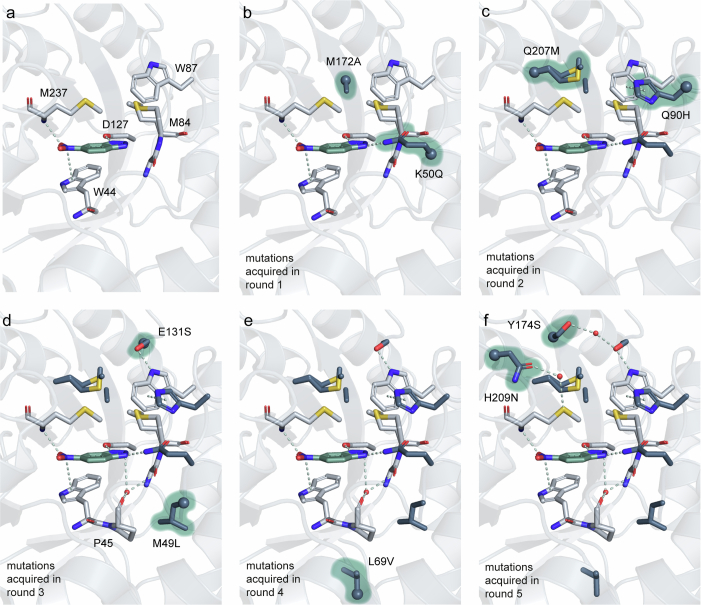
Analysis of the interaction network of mutations acquired over the evolutionary trajectory of HG3.R5. **a**, Snapshot of the configuration of the active site of HG3. **b**, In the first round of evolution, mutations M172A and K50Q were acquired, which are in direct contact with the substrate 5-nitrobenzisoxazole (**1**). **c**, In evolution round 2, mutation Q207M complements the prior mutation M172A, while mutation Q90H allows for the formation of a π-stacking interaction with W87. **d**, In evolution round 3, mutation E131S allows the formation of a hydrogen bond with W87, and mutation M49L opens up space close to P45, which is observed to substantially shift position in the final variant HG3.R5. P45’s conformational change presumably leads to the binding of water in the active site, which contributes to the stabilization of the transition state. **e**, In evolution round 4, the acquisition of mutation L69V seems to further assist in the observed conformational change of P45. **f**, In the final evolution round, mutations H209N and Y174S are introduced, which further anchor M237 and S131 via an elaborate water network.

**Extended Data Fig. 6:**
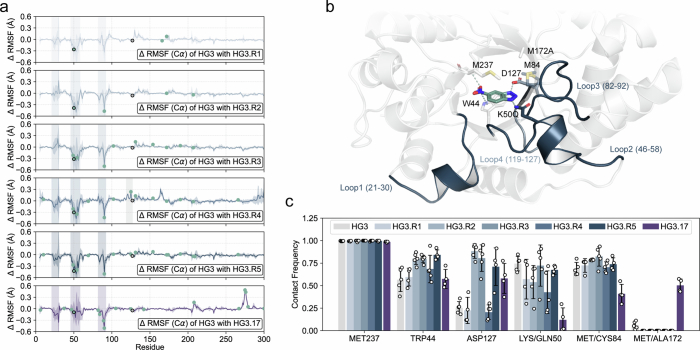
MD simulations of HG3 and its variants. Each variant was simulated 5 times with the substrate 5-nitrobenzisoxazole (**1**) for 100 ns recording every 1000 steps (step size = 2.0 fs), resulting in a total of 250000 data points per variant. **a**, Root-mean-squared fluctuation difference against HG3. Mutations acquired during the evolution are denoted by green markers, while the catalytically active D127 and the K50Q mutations are highlighted by outlined spheres. Three distinct regions exhibiting heightened rigidity (21–30, 46–58 and 82–92) are emphasized with shading. In HG3.R4, a region encompassing D127 (119–127) experiences a loss of rigidity, as indicated by a gray-shaded area. Rigidity is subsequently restored in HG3.R5. **b**, Crystal structure of HG3.R5 highlighting active site residues and the rigidifying regions (loop 1–loop 4). Loop 2 - comprising the K50Q mutation - gains rigidity compared to HG3 over the course of evolution, while loop 4 - comprising the catalytic active D127 temporarily - gains flexibility in HG3.R4. **c**, Contact frequencies of active site residues with the substrate 5-nitrobenzisoxazole (**1**). The data are shown from n = 4 (HG3.R1, HG3.R5 and HG3.17) or n = 5 (HG3, HG3.R2, HG3.R3 and HG3.R4) simulations and presented as mean ± SD. Source data

In HG3.R5, mutations acquired in the first round, such as M172A and K50Q, directly contact the substrate (Extended Data Fig. 5b), while subsequent substitutions fan outward from the active site in an intricate interaction network that optimized ligand placement relative to the catalytic machinery (Extended Data Figs. 5 and 6). In this context, comparing the root mean square fluctuation (RMSF) per residue of HG3.R1–R5 variants with the structure of in silico designed HG3 revealed that three distinct protein regions (21–30, 46–58 and 82–92) exhibited a gradual gain in rigidity during evolution (Extended Data Fig. 6a,b). These rigidifying regions, which line one side of the active site tunnel and include the mutation K50Q, may modulate the contact frequencies of Q50 with the substrate 5-nitrobenzisoxazole (**1**) (Extended Data Fig. 6c). Noteworthy in this context is a 2.5 Å shift of the indole moiety of W87 toward the ligand, which brings it close to Q50 in HG3.R5. Notably, HG3.17 features a similar movement of the W87 side chain, albeit accompanied by a tryptophan ring flip^35^ (Fig. 4d,e).

### Fitness landscape

**Fig. 5:**
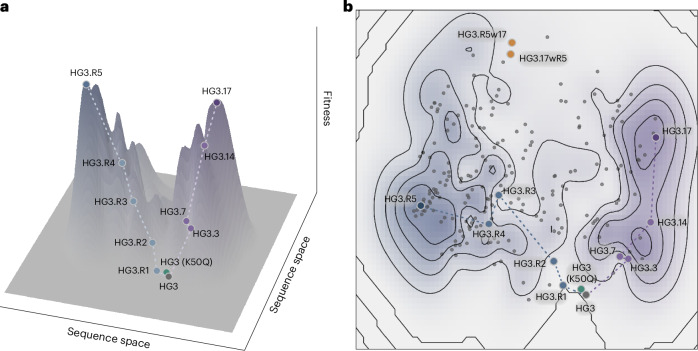
Schematic representation of HG3’s fitness landscape. Overall, 208 unique sequence–function pairs stemming from the evolutionary trajectories of HG3.R5 and HG3.17 as well as combinatorial variants generated via gene shuffling were used to map HG3’s fitness landscape. For each variant, the underlying sequence space is represented by a principal component analysis of the embeddings extracted from the ESM algorithm^39^. The fitness values represent the relative activity of each variant versus HG3.R5 calculated from the activity measurements of three biological replicates under screening assay conditions. The activity data of HG3.3, HG3.7 and HG3.14 were inferred from literature values^21^. **a**, Three-dimensional representation of the fitness landscape. **b**, Topological view of the fitness landscape showing all data points. Sequence–activity pairs derived from the shuffled libraries are highlighted as gray dots (195 variants). Sequence–activity pairs derived from the HG3.R5 trajectory are highlighted as blue dots and labeled with their alphanumerical identifiers (five variants), while sequence–activity pairs from the HG3.17 trajectory are highlighted as purple dots and labeled with their alphanumerical identifiers (four variants). Combined variants HG3.R5w17 and HG3.17wR5 are highlighted as orange dots and HG3 K50Q is highlighted as a green dot. The in silico designed HG3 is highlighted with a large gray dot. Source data

To characterize the fitness landscape surrounding the HG3.R5 and HG3.17 variants, we investigated whether their respective mutations could complement each other. We constructed two combined variants, called HG3.R5w17 and HG3.17wR5, by incorporating all additional mutations found in the other improved Kemp eliminase (w17 and wR5, respectively) into HG3.R5 and HG3.17 (Fig. 3c). Surprisingly, both combined enzymes retained Kemp elimination activity, although they exhibited substantially reduced *k*_cat_ values (Table 1)_._ Intrigued by these results, we shuffled the HG3.R5 and HG3.17 genes in varying ratios. Subsequent analysis of active variants found in the shuffled libraries, complemented with data for variants from the HG3.R5 and HG3.17 evolution trajectories, resulted in 208 unique sequence–function data points. The underlying fitness landscape defined by these data points was elucidated by a principal component analysis of each variant’s embedding vector, which was derived from the evolutionary scale modeling (ESM) algorithm^39^ (Fig. 5). This analysis revealed a deep valley between the fitness peaks representing the most improved variants, HG3.R5 and HG3.17, with no discernible ridge connecting the sequences. Overall, this empirically deduced fitness landscape indicates that the optimized variants, which are 29 mutations apart, are evolutionarily more distant from each other than from the parental sequence HG3.

## Discussion

The fitness landscape is a nearly century-old foundational concept in evolutionary biology^40^. Today, it is accepted that the shape of this genotype–phenotype fitness map defines evolvability^41^ and that relatively few mutational paths lead to fitter proteins^42^. In analogy to natural evolution, the laboratory optimization of enzymes selects high-fitness genotypes and does not usually allow an enzyme to traverse low-fitness valleys between local peaks of intermediate fitness and higher fitness peaks nearby^43^.

While the way sequence maps function does not change, our results indicate that protein sequence space can be more effectively searched by reducing the number of possible evolutionary pathways through the removal of destabilizing mutations. The resulting ‘condensed’ search space increases the probability of finding evolutionarily accessible mutational paths that can be effectively navigated by the stepwise introduction and combination of single-site mutations in the climb to higher ground. Using this strategy, we obtained an artificial biocatalyst that accelerates proton transfer with true enzymatic efficiency (*k*_cat_/*k*_uncat_ = 6.1 × 10^8^)^23^ within a remarkably short span of five evolution rounds. Notably, in analogy to natural evolution, each design-built-test cycle reconsidered all amino acid sites predicted to be nondestabilizing, enabling the escape from local maxima by permitting further modifications or reversal of specific mutations.

Evolutionary predictability is a fundamental question in biology and the topic of a long-running debate, reflecting relatively limited empirical data for real fitness landscapes^41^. While a few studies have provided evidence for convergence^44–46^ (the independent evolution of the same or a similar outcome), other experiments have highlighted how evolutionary trajectories are dependent on random events^47,48^. Interestingly, careful analysis of these experiments revealed that the degree of parallelism seems to vary according to the organizational level—shared adaptations at the level of genes and metabolic pathways are more common than shared substitutions of nucleotides^41,44^.

Our results show that although distinct in sequence, the optimized designer enzyme HG3.R5 harnesses the same catalytic principles exploited in other Kemp eliminases^21,35^. This bodes well for enzyme engineers as it indicates that individual protein scaffolds can house several, equally viable solutions for the same catalytic problem. The existence of more than one proverbial ‘needle in the haystack’ increases the chances of developing highly active catalysts using the experimental and in silico tools in hand today.

## Methods

### Materials

Commercial reagents are listed in Supplementary Data 2. Oligonucleotides (except for oligo pools) were ordered from Microsynth. Clonal genes and oligo pools were ordered from Twist Bioscience.

### Synthesis of 5-nitrobenzisoxazole (1)

The synthesis of 5-nitrobenzisoxazole (**1**) was performed according to published protocols^51^. 1,2-Benzisoxazole (5 ml, 5.87 g) was added to concentrated H_2_SO_4_ (20 ml) at 0 °C until the solution turned yellow. A mixture of concentrated HNO_3_ (3.4 ml) and concentrated H_2_SO_4_ (1.3 ml) was added slowly at 0 °C, and the solution was stirred for 30 min. The reaction product was poured onto an ice/water mixture (1:1, 100 ml), and the crystals that formed were collected by filtration, washed with ice-cooled water and dried. The crude product was purified by normal-phase flash chromatography (RediSep column) with cyclohexane and ethyl acetate as mobile phases. The solvent was removed to yield 5-nitrobenzisoxazole (**1**) (3.05 g) as colorless needles (^1^H NMR (500 MHz, CDCl_3_) *δ* = 8.90 (d, 1 H), 8.73 (d, 1 H), 8.51 (dd, 1 H), and 7.76 (d, 1 H) and ^13^C NMR (500 MHz, CDCl_3_) *δ* = 164.4, 147.1, 144.8, 125.6, 121.9, 119.2, and 110.5).

### Plasmid constructs

The HG3 and HG3.17 constructs were ordered from Twist Bioscience as cloned genes in a pET28b vector between the NcoI/XhoI restriction sites containing a C-terminal His_6_-tag with a stop codon. The pelB-HG3 and pelB-HG3.17 variants used for screening were obtained by cloning the genes into the pET22b vector using NcoI/XhoI restriction enzymes, introducing an N-terminal pelB signal peptide and a C-terminal His_6_-tag. Sequences of all genes are reported in the Supplementary Information.

### Generation of complex single-site libraries using oligo pools

The cloning procedure is illustrated in Extended Data Fig. 1.

The oligo pool from Twist Bioscience was resuspended in MilliQ-water to yield a DNA concentration of 5 ng µl^−1^. The pool was amplified by PCR (25 µl) using KAPA HiFi HotStart DNA polymerase according to the manufacturer’s protocol, with the common forward and reverse primers with a 2 µl oligo pool. The individual oligo subpools were amplified analogously with subpool-specific primers (designed with LibGENiE^19^) and 1 µl purified oligo pool amplification product (~50 ng). The flanking upstream and downstream regions of the fragments were amplified using the parent variant as a template and corresponding primers (designed with LibGENiE^19^) in combination with T7_fw (TAATACGACTCACTATAGGG) or T7_rv (TGCTAGTTATTGCTCAGCGG).

Full genes were reassembled by an overlap extension PCR (25 µl) with KAPA HiFi HotStart DNA polymerase according to the manufacturer’s protocol. In total, 1 µl amplified oligo subpool and 1 µl upstream and downstream PCR were used as template fragments with the T7_fw and T7_rv primers. The protocol included eight amplification cycles without flanking T7 primers, followed by 25 cycles with T7 primers.

The reassembled genes were introduced into the pET22b vector containing the pelB signal tag using the MEGAWHOP^52^ cloning strategy. The PCR mixture (50 µl) contained 10 µl 5× Q5 reaction buffer, 2 µl dNTPs (10 mM each), 6 µl purified overlap extension PCR product (50–150 ng µl^−1^), 1 µl Q5 high-fidelity DNA polymerase and 1 µl template plasmid (100 ng, pET22b-pelB containing the parent gene of the evolution round). The template DNA was digested by incubation with DpnI (37 °C for 2 h).

### Generation of combinatorial libraries

The cloning procedure is illustrated in Extended Data Fig. 2.

Primers encoding beneficial mutations and the parent amino acid were ordered from Microsynth. If beneficial mutations had appeared at the same site or in close proximity, they were encoded combinatorically on primers spanning the corresponding region using degenerate codons if feasible. Primers for such regions were mixed in equimolar quantities, ensuring an even distribution of the mutations.

Gene fragments spanning primer regions were produced with complementary overlaps. The PCR (50 µl) contained 10 µl 5× Q5 reaction buffer, 1 µl dNTPs (10 mM each), 1 µl template plasmid (50–100 ng µl^−1^ of pET22b-pelB containing the parent gene for the evolution round), 2.5 µl combinatorial primer mix (10 µM), 2.5 µl complementary overlap primer and 1 µl Q5 high-fidelity DNA polymerase.

The genes were reassembled by an overlap extension PCR (50 µl) with Q5 high-fidelity DNA polymerase according to the manufacturer’s protocol. The reaction contained 1 µl of each purified fragment as a template and 2.5 µl of the T7_fw and T7_rv primers, respectively. The temperature protocol included eight amplification cycles without flanking T7 primers, followed by 25 cycles including the flanking T7 primers.

The PCR product was cloned in a pET22b-pelB vector using restriction enzymes XbaI and XhoI followed by a ligation step with T4 ligase. All procedures were carried out according to the manufacturer’s protocols.

### Generation of shuffled libraries

The genes of HG3.17 and HG3.R5 were amplified from the pET22b-pelB plasmid with T7_fw and T7_rv primers using the Q5 high-fidelity DNA polymerase according to the manufacturer’s protocol. The purified PCR products were mixed in equal quantities (500 ng each) and digested with 5 mU of DNAseI in 50 µl buffer (20 mM MnCl_2_ and 100 mM Tris (pH 7.4)) for 2, 3 and 5 min at 15 °C. DNAseI was inactivated (80 °C for 10 min), and the reactions were purified. In total, 4 ng of digested gene fragments were reassembled with a step-down PCR (50 µl) without primers using the Q5 high-fidelity DNA polymerase according to the manufacturer’s protocol. Reassembled genes were introduced into a pET22b vector containing the pelB signal tag with MEGAWHOP^52^ cloning as described earlier.

### Screening of the single-site variant, hit combination and shuffled libraries

Chemically competent *E. coli* NEB10 cells were transformed with 5 µl of cloning products. Colonies were grown on LB agar plates (100 mg l^−1^ ampicillin). All colonies were scraped off the plate, and plasmids were isolated with the NucleoSpin plasmid kit. Chemically competent *E. coli* BL21 (DE3) cells were transformed with 5 µl of the isolated plasmid and grown on LB agar plates (100 mg l^−1^ ampicillin). Precultures were prepared by inoculating 1 ml LB medium (100 mg ml^−1^ ampicillin) in 96-well deep-well plates (DWP) with single *E. coli* BL21 colonies and grown overnight at 30 °C with 300 rpm in a Duetz-System (Adolf Kühner AG). In total, 2 µl of the preculture was used to inoculate 1 ml of ZYM-5052 autoinduction medium (100 mg ml^−1^ ampicillin) in a 96-well DWP. The main cultures were grown for 15 h at 30 °C and 24 h at 20 °C at 300 rpm in a Duetz-System.

The activity assay was performed in 96-well microtiter plates. Main cultures were diluted into reaction buffer (50 mM sodium phosphate (pH 7) and 100 mM NaCl) to obtain 100 µl of final volume. The ratio of culture to reaction buffer was adjusted according to the evolution round (R1: 20:80; R2: 20:80; R3: 10:90; R4: 1:99; R5: 0.5:99.5). The substrate (25 mM 5-nitrobenzisoxazole (**1**) in acetonitrile) was diluted into the reaction buffer (1:50), and 100 µl of this solution was immediately transferred to the diluted main cultures, initiating the reaction in a total volume of 200 µl containing 250 µM 5-nitrobenzisoxazole (**1**), 1% acetonitrile and round dependent volume of grown cultures (R1, 10 µl; R2, 10 µl; R3, 5 µl; R4, 0.5 µl and R5 0.25 µl). The reaction was monitored at 380 nm and 35 °C on a Tecan Spark (Tecan Group).

### Production and purification of the HG3 variants

Genes were cloned into the pET28b plasmid between NcoI/XhoI restriction sites (without the pelB-leader sequence but with the C-terminal His_6_-tag and stop codon). A preculture of 6 ml LB medium (50 µg ml^−1^ kanamycin) was grown overnight at 37 °C. In total, 500 ml of LB medium (50 µg ml^−^^1^ kanamycin) was inoculated with 5 ml preculture and incubated at 37 °C. Production of the HG3 protein was induced when the optical density at 600 nm (OD_600_) reached a value of 0.6 by adding 250 µM isopropyl-β-d-thiogalactopyranoside. The culture was then incubated overnight at 18 °C.

Cells were collected by centrifugation for 60 min at 3,700*g*, 4°C, resuspended in 15 ml buffer (50 mM Tris–HCl (pH 7.4) and 500 mM NaCl), lysed by ultrasonic treatment (amplitude, 50%; pulse, 1 s/1 s and time, 1 min; Sonoplus) and clarified by centrifugation (1 h at 21,000*g*) as well as filtration (0.45 µm). The lysate was purified via nickel affinity chromatography on an ÄKTA Pure FPLC system (GE Healthcare) using a 5 ml HisTrap FF crude column (Cytiva). Loading buffer consisted of 500 mM NaCl and 20 mM imidazole in 50 mM Tris–HCl (pH 7.4), while the elution buffer contained 500 mM NaCl and 300 mM imidazole in 50 mM Tris–HCl (pH 7.4). Samples were desalted with three 5 ml HiTrap desalting columns (Cytiva) into a buffer containing 5 mM sodium phosphate (pH 6.0) and 20 mM NaCl and concentrated by ultrafiltration (Amicon Ultra-4 10K; Merck Millipore).

### Progress curves

Progress curves to obtain kinetic parameters were recorded according to the protocol given in ref. ^35^. The 10× concentrated Kemp eliminase variant stocks were prepared in reaction buffer (55.5 mM sodium phosphate (pH 6.8) and 111.1 mM NaCl), and 10× concentrated stocks of 5-nitrobenzisoxazole (**1**) were prepared in methanol. In total, 120 μl of reaction buffer and 15 μl of the enzyme stock solution were added to a quartz cuvette, and the absorption at 440 nm in a Cary 60 UV–Vis spectrophotometer (Agilent Technologies) was set to zero. The assay was initiated by the addition of 15 μl 10× concentrated substrate stock. The final enzyme concentration in the reaction buffer varied according to the variant (HG3, 19.0 μM; HG3.17, 61.2 mM; HG3.R1, 9.02 μM; HG3.R2, 2.23 μM; HG3.R3, 1.33 μM; HG3.R4, 225 nM; HG3.R5, 126 nM; HG3.R5w17, 3.88 μM and HG3.17wR5, 3.70 μM).

Progress curves were measured by observing product formation at 440 nm (25 °C), applying an extinction coefficient of 1,050 M^−1^ cm^−1^. The initial substrate concentration was evaluated by diluting the reaction in an alkaline solution and measuring the product absorbance at 380 nm, applying an extinction coefficient of 15,800 M^−1^ cm^−1^ (15 μl reaction mixture was diluted in 235 μl of a 0.68 M sodium hydroxide solution). The data were analyzed using KinTek Explorer (6.1)^53,54^ to extract the steady-state parameters (Supplementary Figs. 1a and 2a).

The rate constant for the background reaction (*k*_1_) was fixed to the value reported in the literature (*k*_1_ = 1.04 × 10^−4^)^35^. The reaction rate constants for HG3 and HG3.17 were corrected for a conformational selection step as reported in ref. ^35^ (*k*_2_^HG3^ = 2.32 × 10^−4^ s^−1^, *k*_−2_^HG3^ = 8.12 × 10^−5^ s^−1^, *k*_2_^HG3.17^ = 16.7 × 10^−4^ s^−1^ and *k*_−2_^HG3.17^ = 6.68 × 10^−5^ s^−1^). These reaction rates (*k*_2_ and *k*_−2_) were not available for other variants, which were assumed to be fully active as isolated to avoid overestimation of their activity. Furthermore, the on-rates for substrate and product (that is, *k*_3_ and *k*_−5_, respectively) were fixed to a diffusion-limited value of 1,000 μM^−1^ s^−1^. This left three parameters for fitting (that is, *k*_−3_, *k*_4_ and *k*_5_). The individual microscopic rate constants cannot be determined by fitting, but the values for *k*_cat_ and *K*_m,substrate_ can be estimated reliably using the equations below:

1 $${k}_{{\mathrm{cat}}}=\frac{\,{k}_{4}{k}_{5}}{{k}_{4}+{k}_{-5}+{k}_{5}}=\frac{\,{k}_{4}{k}_{5}}{{k}_{4}+{k}_{5}}$$

2 $${K}_{{\mathrm{m}},{\mathrm{substrate}}}=\frac{\,{k}_{4}{k}_{5}+{k}_{-3}({k}_{-4}+{k}_{5})}{\left(\frac{{k}_{2}{k}_{3}}{{k}_{-2}+{k}_{2}}\right)({k}_{4}+{k}_{-4}+{k}_{5})}=\frac{\,{k}_{4}{k}_{5}+{k}_{-3}{k}_{5}}{\left(\frac{{k}_{2}{k}_{3}}{{k}_{-2}+{k}_{2}}\right)({k}_{4}+{k}_{5})}$$

3 $${K}_{{\mathrm{m}},{\mathrm{substrate}}}=\frac{\,{k}_{4}{k}_{5}+{k}_{-3}(\,{k}_{-4}+{k}_{5})}{\,{k}_{3}({k}_{4}+{k}_{-4}+{k}_{5})}=\frac{\,{k}_{4}{k}_{5}+{k}_{-3}{k}_{5}}{\,{k}_{3}({k}_{4}+{k}_{5})}$$

### Initial rate analysis

The *k*_cat_/*K*_m_ values shown in Table 1 were determined using initial rates analysis for product formation. Reactions were initiated by adding enzyme (HG3, 750 nM; HG3.R1, 459 nM; HG3.R2, 112 nM; HG3.R3, 65.7 nM; HG3.R4, 25.1 nM; HG3.R5, 6.6 nM; HG3.17, 6.8 nM; HG3.17wR5, 209 nM; HG3.R5w17, 208 nM) to the benzisoxazole substrate (31.5 µM to 2.0 mM final concentration) in 10% methanol, 100 mM NaCl and 50 mM sodium phosphate buffer (pH 7). Product formation was monitored at 380 nm with *ε*_380_ (pH 7) = 15,784.9 M^−1^ cm^−1^ (${\varepsilon }_{380}={\varepsilon }_{\max }/\left(1+{10}^{\left({{\rm{p}}K}_{a}-{\rm{pH}}\right)}\right)$; *ε*_max_ = 15,800 M^−1^ cm^−1^) in a Cary 60 ultraviolet/visible spectrometer (Agilent Technologies) at 27 °C using glass cuvettes (1 cm path length). Data were fitted to the linear portion of the Michaelis–Menten model (v_0_ = (*k*_cat_/*K*_m_)[E_0_][S]), and *k*_cat_/*K*_m_ was deduced from the slope. The substrate stock solution concentration was determined by preparing a 2000-fold dilution in 10% methanol containing 10 mM sodium hydroxide and measuring the sample absorption at 380 nm with *ε*_380_ (pH > 9) = 15,800 M^−1^ cm^−1^.

### Melting temperature determination

The melting temperature of the purified variants was determined with a Prometheus Panta (NanoTemper Technologies). The proteins were prepared at approximately 1 mg ml^−1^ in 5 mM sodium phosphate buffer (pH 6) containing 20 mM NaCl. The thermal unfolding experiment was performed from 20 to 95 °C with a ramp of 1 °C min^−1^. The derived melting profile was analyzed using the accompanying software (PR.Panta Analysis v1.6.3).

### Crystallization of HG3.R5 with the TSA 6-nitrobenzotriazole (3)

HG3.R5 was produced and purified as described above, but the desalting step was performed on a size-exclusion column (HiLoad 16/600 Superdex 75 prep grade) with a buffer containing 5 mM sodium phosphate (pH 6) and 20 mM NaCl. The fractions containing protein were concentrated by ultrafiltration (Amicon Ultra-15 10K; Merck Millipore). The protein solution was prepared at a concentration of 30 mg ml^−1^ containing 5 mM 6-nitrobenzotriazole (**3**) (from a 100 mM stock solution in DMSO), resulting in a solution containing 5% DMSO, 4.75 mM sodium phosphate at pH 6 and 19 mM NaCl. Crystallization was performed using the sitting drop vapor diffusion method at 20 °C in Intelli-Plates 96-3 LVR (Hampton Research) at the Protein Crystallization Center (University of Zurich, Switzerland). A 150 nl drop of enzyme and substrate solution (30 mg ml^−1^ enzyme with 5 mM 6-nitrobenzotriazole (**3**)) was mixed with 150 nl precipitants (100 mM Tris–acetate (pH 7.4) and 1.3 M (NH_4_)_2_SO_4_) and equilibrated against a 50 µl reservoir solution (100 mM Tris–acetate (pH 7.4) and 1.3 M (NH_4_)_2_SO_4_). Crystal formation was observed after 25 days of incubation, and crystals were picked after incubation for a total of 41 days. In total, 2 µl of 30% glycerol in a reservoir solution was added to the drop as a cryoprotectant. The crystals were snap-frozen and stored in liquid nitrogen.

Diffraction data were collected at the Swiss Light Source (Paul Scherrer Institute, Switzerland) on the beamline X06SA-PX at a temperature of 100 K and wavelength of 1.0000 Å. The data were (1) indexed and integrated with XDS (version 10 January 2022), (2) scaled and merged with Aimless (v0.7.9) and (3) solved using the experimental Kemp structure PDB code 5RGA (chain A) by molecular replacement using MOLREP (v11.0)^55^. Iterative refinement and manual model building steps were performed with REFMAC (v5.8.0419)^56^ and Coot (v0.9.8.92)^57^, respectively, embedded in the CCP4i2 (v8.0.013)^58^ suite. The final model with a resolution of 1.5 Å and a MolProbity^59^ score of 1.13 contained two protein chains (A and B) including the TSA (**3**).

### Library design based on evolutionary information

An initial multiple sequence alignment (MSA) was created with the online HHblits tool^60^ (https://toolkit.tuebingen.mpg.de/tools/hhblits), using the UniRef30_2022_02 database and default parameters. The MSA was further processed with HHfilter^61^ (https://toolkit.tuebingen.mpg.de/tools/hhfilter) with the following settings: max ident: 90; min seq ident: 30; rest default. Variants were selected based on the consensus and frequency strategy outlined in the HotSpot Wizard overview^30^, leading to 76 additional variants included in each single-site library.

### Library design based on computational mutational scanning

ΔΔ*G* predictions were based on a protocol published by the official Rosetta forums (https://www.rosettacommons.org/node/11126, 10 February 2021), and the relevant Python script can be found in the Supplementary Information. Each variant was predicted three times, and the lowest energy obtained was compared to the wild-type energies to calculate differences in free energy (Python version 3.8, PyRosetta version available at PyRosetta4.Release.python38.linux r275 2021.07+release.c48be26 c48be2695c4ba637c6fa19ee5d289fd9a8aa99ef).

### Library design based on tunnel and ligand analysis

Enzyme tunnel analysis was performed with CAVER 3.0 (ref. ^49^) using the following parameters: probe_radius 0.9, shell_radius 4.0, shell_depth 5.0, frame_weighting_coefficient 1.0 and frame_clustering_threshold 1.0. The TSA (**3**) was introduced by aligning the crystal structure of HG3.17 (PDB 4BS0) to an AlphaFold^50^ model of each round’s parent variant. Sites for randomization were selected based on distances to the ligand and tunnels.

### Fitness landscape

The protein sequences were encoded using the esm.pretrained.esm2_t48_15B_UR50D() model^39^. After extracting each variant’s embedding, these were reduced in dimensionality through principal component analysis (python package: sklearn, default settings, *n*_components = 2). In the absence of experimental data, the space outside the measured variants was set to 0. Within the area of measured variants, we performed radial basis function interpolation (python package: scipy, smooth = 0.1) and applied a further filter (python package: scipy.ndimage.uniform_filter, size = 2, mode = ‘constant’) to reduce ruggedness.

### MD simulation of HG3 variants

The crystal structures of HG3.R5 (PDB 8RD5) were used for the homology modeling of HG3.R1, HG3.R2, HG3.R3 and HG3.R4. Simulations were also carried out with HG3 (PDB 5RGA) and HG3.17 (PDB 5RGE).

The cocrystallized TSA (**3**) was manually replaced in all variants by the substrate 5-nitrobenzisoxazole (**1**). The PARSE force field was used at a pH 7, and protonation states for the titratable amino acids of all variants were determined using PROPKA3 (ref. ^62^), except for catalytic base D127 which was set up deprotonated.

Substrate parameters were generated using Ambertools (23.3) packages antechamber and parmchk2 (ref. ^63^) with bond charge correction (AM1-BCC). The resulting mol2 and frcmod files were converted into xml files using ParmEd (4.1.0). The simulations were performed using OpenMM (8.1.0)^64^ with the ff14SB force field^65^. The rectangular box with a padding of 1.0 nm was solvated with water (TIP3P water model), and the total charge was neutralized. Energy minimization was conducted until 10 kJ mol^−1^ tolerance energy was reached. A temperature of 308.15 K and a pH of 7 were set, and the Langevin integrator was used with a friction coefficient of 1 ps^−1^ and a step size of 2.

The long-range Coulomb interactions were computed by the particle mesh Ewald method, with a cutoff of 1 nm for the direct space interactions. The solvent was equilibrated by 5 ns NVT equilibration (using a Langevin integrator for temperature coupling) followed by 5 ns pressure coupled NPT equilibration using a Monte Carlo barostat at 1 atm pressure and 308.15 K reference temperature. The protein backbone and the substrate were restrained with a force of 100 kJ mol^−1^ Å^−2^. Finally, all restraints were removed, and a free equilibration of 5 ns was performed. The resulting system was used to produce five replicates of 100 ns each, with periodic boundary conditions. Every 1,000 steps, the trajectories were recorded.

Analysis was conducted using MDTraj^66^. RMSF of the backbone Cα was calculated by superposing all frames onto the first frame as a reference.

Contact frequency between protein and 5-nitrobenzisoxazole (**1**) atoms was calculated with the ContactTrajectory function of the MDTraj (1.9.9)^66^ derived Contact Map Explorer package. All substrate atoms were used as queries, while the full protein served as a haystack with a cutoff distance of 3 Å. Replicates in which the substrate left the binding site during simulation were not considered for this analysis.

### QM calculations

Restricted geometry optimizations and transition state searches were carried out with Gaussian 16 (ref. ^67^) on cluster models. Cluster models were generated from chain A of the crystallographic structure of HG3.R5 (PDB 8RD5) in complex with the TSA (**3**) and replaced with fully optimized 5-nitrobenzisoxazole (**1**). Cluster models include the substrate and the following residues: W44, P45, N47 (backbone only), S48 (backbone only), L49 (backbone only), Q50, G82, and G83 and D127 (side chain up to Cβ). All C atoms except substrate positions 3, 3a and 7a were kept fixed at the crystallographic coordinates. Calculations were carried out using the M06-2X^68^ hybrid functional and 6-31G(d) basis set with ultrafine integration grids. Solvent effects in water were considered implicitly through the IEF-PCM polarizable continuum model^69^. The energies of the calculated structures are summarized in Supplementary Table 2.

### QM/MM calculations

The initial geometry for the HG3.R5–5-nitrobenzisoxazole (**1**) complex was generated from chain A of the crystallographic structure (PDB 8RD5) of HG3.R5 in complex with the TSA (**3**) and replacing its coordinates with the optimized coordinates of 5-nitrobenzisoxazole (**1**) (M06-2X/6-31G(d) level of theory). Crystallographic waters were preserved. The enzyme–substrate complex was then prepared for classical MD relaxation with the Amber 22 (ref. ^[63^) suite using the ff19SB^70^ force field for the protein and gaff2 (ref. ^71^) for the substrate. The complex was immersed in a water box with a 10 Å buffer of OPC^72^ water. A two-stage geometry optimization approach was implemented. The first stage minimizes only the positions of solvent molecules, and the second stage is an unrestrained minimization of all the atoms in the simulation cell. The systems were then heated for 100 ps by incrementing the temperature from 0 to 300 K under a constant pressure of 1 atm and periodic boundary conditions with a timestep of 1 fs. Harmonic restraints of 50 kcal mol^−1^ Å^−2^ were applied to the solute, with the Andersen^73^ temperature coupling scheme to control and equalize the temperature. The last geometry of the equilibration trajectory was extracted, and solvent molecules were trimmed, leaving an 8 Å thick water shell around the solute.

Full geometry optimizations and transition state searches were carried out with Gaussian 16 (ref. ^67^; ONIOM^74^ QM/MM method, with electrostatic embedding) using the M06-2X hybrid functional and 6-31+G(d) basis set with ultrafine integration grids for the QM part. The MM part was described with the ff14SB^65^, gaff2 (ref. ^71^) and TIP3P^75^ force fields for the protein, ligand and water, respectively. The QM layer includes the whole 5-nitrobenzisoxazole (**1**), the side chains (up to Cβ) of D127 and Q50 and the two conserved water molecules shown in Extended Data Fig. 4. Only solvent molecules were kept frozen, except for the two water molecules in the QM layer, to allow adaptation of the enzyme along the reaction path. All stationary points were characterized by a frequency analysis performed at the same level of theory, from which thermal corrections were obtained at 298.15 K. ONIOM energies, entropies, enthalpies, Gibbs free energies and lowest frequencies of the calculated structures are summarized in Supplementary Table 3. The effect of crystallographic water *w*_*e*_ on the activation barrier was computed by manually removing *w*_*e*_ and reoptimizing the reactant and transition state at the same level of theory.

### Reporting summary

Further information on research design is available in the Nature Portfolio Reporting Summary linked to this article.

## Online content

Any methods, additional references, Nature Portfolio reporting summaries, source data, extended data, supplementary information, acknowledgements, peer review information; details of author contributions and competing interests; and statements of data and code availability are available at 10.1038/s41589-024-01712-3.

## Supplementary information

Supplementary Information Supplementary Figs. 1 and 2, Supplementary Tables 1–3 and Supplementary Note (amino acid and nucleotide sequences, cartesian coordinates of cluster model QM structures and Python script for ΔΔ*G* predictions with PyRosetta).

Reporting Summary

Supplementary Data 1 Supporting data for Supplementary Figs. 1 and 2.

Supplementary Data 2 List of commercial reagents.

## Source data

Source Data Fig. 1 Data used to create Fig. 1c,d.

Source Data Fig. 2 Data used to create Fig. 2b,d,e.

Source Data Fig. 3 Data used to create Fig. 3d.

Source Data Fig. 4 Data used to create Fig. 4b,c.

Source Data Fig. 5 Data used to create the 3D plot of the fitness landscape.

Source Data Extended Data Fig. 3 Data used to create Extended Data Fig. 3b.

Source Data Extended Data Fig. 6 Data used to create Extended Data Fig. 6a,c.

## Data Availability

The data that support the findings of this work are available in this article, Extended Data Figs. 1–6, Source Data, Supplementary Data 1 and 2 and Supplementary Information. Crystallographic coordinates of the binary complex of HG3.R5 have been deposited in the PDB as 8RD5. The HG3 and HG3 variant crystal structures used in molecular replacement, preparation of comparative figures, MD and QM/MM experiments can be accessed via PDB codes 5RGA, 5RGE, 8RD5, 4BS0, 7K4Q and 7K4Z. MD trajectories, computed geometries and energies can be accessed through the Zenodo repository at 10.5281/zenodo.12756879 (ref. ^76^). Source data are provided with this paper.

## Code availability

The Python script to make ΔΔ*G* predictions can be found in the Supplementary Information. Data and scripts used to build the protein fitness landscapes can be accessed via https://github.com/ccbiozhaw/FitLan.
