# Supplementary material for: Enriching productive mutational paths accelerates enzyme evolution
Source: Nat Chem Biol. 2024 Sep 11;20(12):1662–9. doi: 10.1038/s41589-024-01712-3 (PMC11581979; doi:10.1038/s41589-024-01712-3)
Supplement: Supplementary file 1 — Supplementary Figs. 1 and 2, Supplementary Tables 1–3 and Supplementary Note (amino acid and nucleotide sequences, cartesian coordinates of cluster model QM structures and Python script for ΔΔG predictions with PyRosetta). [file 41589_2024_1712_MOESM1_ESM.pdf]

nature chemical biology

Supplementary information https://doi.org/10.1038/s41589-024-01712-3

Enriching productive mutational paths accelerates enzyme evolution

In the format provided by the authors and unedited

Table of contents:

Supplementary Fig. S1 | Progress curves of product formation. 2

Supplementary Fig. S2 | Progress curves of product formation. 3

Supplementary Table S1 | X-ray crystallographic data collection and refinement statistic 4

Supplementary Table S2 | Energies of cluster model QM optimized structures. 5

Supplementary Table S3 | Energies, entropies, and lowest frequencies of QM/MM optimized structures 6

Amino acid sequences of the HG3 variants 7

Nucleotide sequences of HG3 variants 9

Cartesian coordinates of cluster model QM structures calculated with PCM(H2O)/M06-2X/6-31G(d) 12

Python script for ΔΔG predictions with PyRosetta 28

SI References 32

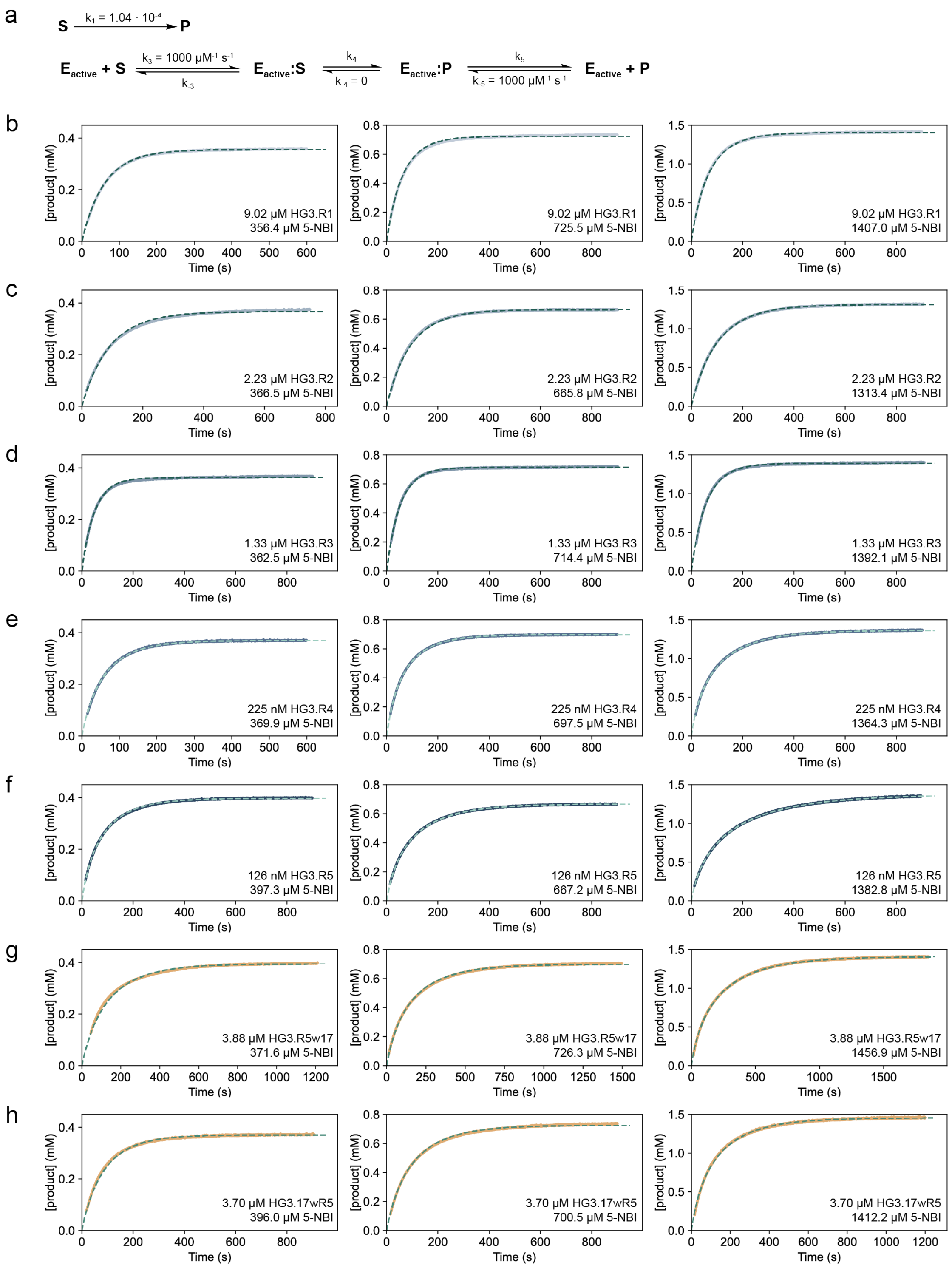

Supplementary Fig. S1 | Progress curves of product formation. (a) The kinetic scheme used for fitting the progress curves including the spontaneous product (5-NBI, 5-nitrobenzisoxazole) formation. Numeric fitting of the total time course data for 5-nitrobenzisoxazole conversion by variants HG3.R1 (b), HG3.R2 (c), HG3.R3 (d), HG3.R4 (e), HG3.R5 (f), HG3.R5w17 (g) and HG3.17wR5 (h). Fitting was performed as described in the Materials and Methods section and the steady-state parameters for k_{cat} and K_m were calculated according to equations E1 and E3.

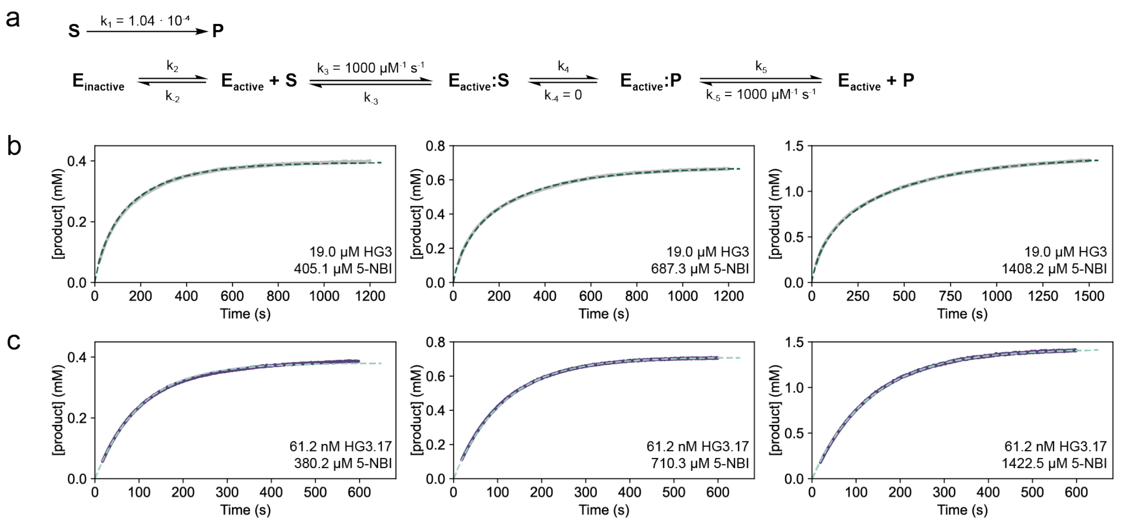

Supplementary Fig. S2 | Progress curves of product formation. (a) The kinetic scheme used for fitting the progress curves including the spontaneous product (5-NBI, 5-nitrobenzisoxazole) formation. Numeric fitting of the total time course data for 5-nitrobenzisoxazole conversion by HG3 (b), HG3.17 (c). The reaction rates determining the conformational selection step (k_1 and k_{-1}) were obtained from Otten et. al.[1] (For HG3: $k_2^{HG3} = 2.32 \cdot 10^{-4}\ s^{-1}$ and $k_{-2}^{HG3} = 8.12 \cdot 10^{-5}\ s^{-1}$; For HG3.17: $k_2^{HG3.17} = 16.7 \cdot 10^{-4}\ s^{-1}$ and $k_{-2}^{HG3.17} = 6.68 \cdot 10^{-5}\ s^{-1}$). The fitting was performed as described in the Materials and Methods section and the steady-state parameters for k_{cat} and K_m were calculated according to equations E1 and E2

Supplementary Table S1 | X-ray crystallographic data collection and refinement statistic

**Kemp variant HG3.R5**<br>Values in () are for highest-resolution shell	
**Data collection**	
Space Group	P 1
Cell dimensions:	
a, b, c (Å)	44.41, 50.07, 65.58
α, β, γ (°)	100.72, 102.70, 99.30
No. unique reflections	81699 (3878)
Resolution (Å)	48.08 – 1.50 (1.50 - 1.53)
R meas	0.079 (0.255)
R pim	0.056 (0.180)
CC1/2	0.995 (0.957)
Mean(I)/sd(I)	12.7 (6.8)
Completeness (%)	96.4 (91.9)
Redundancy	3.3 (3.3)
Wilson B-factor ($Å^2$)	7.1
**Refinement**	
Resolution (Å)	43.20 – 1.50
No. reflections all / free	81698 / 4133
Rwork / RFree	0.103 / 0.142
No. atoms	10071
Protein	9427
Ligand/ion	137
Water	507
B-factor	
Protein	7.88
Ligand/ion	17.65
Water	21.87
R.m.s deviations	
Bond lengths (Å)	0.0117
Bond angles (°)	1.65
Clashscore	3
Ramachandran favoured (%)	98.5
Ramachandran outliers	0
**PDB entry**	**8RD5**

Supplementary Table S2 | Energies of cluster model QM optimized structures.

	E_{QM} (Hartree)[b]
**1**-w_e (QM)	–3534.745450
**1**-w_e TS (QM)	–3534.725458
**1** (QM)	–3458.339108
**1** TS (QM)	–3458.318147
**1**-Q50A-w_e (QM)	–3366.078240
**1**-Q50A-w_e TS (QM)	–3366.056004
**1**-Q50A (QM)	–3289.671991
**1**-Q50A TS (QM)	–3289.648237

[a] 1 Hartree = 627.51 kcal mol^{-1}

[b] Calculated with PCM(H_2O)/M06-2X/6-31G(d).

Supplementary Table S3 | Energies, entropies, and lowest frequencies of QM/MM optimized structures.

	E_{ONIOM} (Hartree)[b]	E_{ONIOM} + ZPE (Hartree)[b]	H (Hartree)[b]	S (cal mol^{-1} K^{-1})[b]	G (Hartree)[b]	Lowest freq. (cm^{-1})	# of imag. freq.
1-we (QM/MM)	–1282.034362	–1243.500511	–1241.213510	9398.5	–1245.678078	8.9	0
1-we TS (QM/MM)	–1282.022539	–1243.494585	–1241.206694	9397.5	–1245.671736	–837.4	1
1 (QM/MM)	–1205.622724	–1167.11442	–1164.829116	9387.2	–1169.289268	9.2	0
1 TS (QM/MM)	–1205.610497	–1167.10768	–1164.822647	9385.0	–1169.281770	–713.1	1

[a] 1 Hartree = 627.51 kcal mol^{-1}. Thermal corrections at 298.15 K.

[b] Calculated with ONIOM(M06-2X/6-31+G(d):ff14SB,gaff2,TIP3P).

Amino acid sequences of the HG3 variants

>HG3
MAEAAQSVDQLIKARGKVYFGVATDQNRLTTGKNAAIIQADFGMVWPENSMKWDATEPSQGNFNFAGADYLVNWAQQNGKLIG
GGMLVWHSQLPSWVSSITDKNTLTNVMKNHITTLMTRYKGKIRAWDVVGEAFNEDGSLRQTVFLNVIGEDYIPIAFQTARAAD
PNAKLYIMDYNLDSASYPKTQAIVNRVKQWRAAGVPIDGIGSQTHLSAGQGAGVLQALPLLASAGTPEVSILMLDVAGASPTD
YVNVVNACLNVQSCVGITVFGVADPDSWRASTTPLLFDGNFNPKPAYNAIVQDLQQGSIEGRGHHHHHH-

>HG3.R1 (K50Q, N166S, M172A)
MAEAAQSVDQLIKARGKVYFGVATDQNRLTTGKNAAIIQADFGMVWPENSMQWDATEPSQGNFNFAGADYLVNWAQQNGKLIG
GGMLVWHSQLPSWVSSITDKNTLTNVMKNHITTLMTRYKGKIRAWDVVGEAFNEDGSLRQTVFLNVIGEDYIPIAFQTARAAD
PSAKLYIADYNLDSASYPKTQAIVNRVKQWRAAGVPIDGIGSQTHLSAGQGAGVLQALPLLASAGTPEVSILMLDVAGASPTD
YVNVVNACLNVQSCVGITVFGVADPDSWRASTTPLLFDGNFNPKPAYNAIVQDLQQGSIEGRGHHHHHH-

>HG3.R2 (K50Q, Q90H, M172A, Q207M)
MAEAAQSVDQLIKARGKVYFGVATDQNRLTTGKNAAIIQADFGMVWPENSMQWDATEPSQGNFNFAGADYLVNWAQQNGKLIG
GGMLVWHSHLPSWVSSITDKNTLTNVMKNHITTLMTRYKGKIRAWDVVGEAFNEDGSLRQTVFLNVIGEDYIPIAFQTARAAD
PNAKLYIADYNLDSASYPKTQAIVNRVKQWRAAGVPIDGIGSMTHLSAGQGAGVLQALPLLASAGTPEVSILMLDVAGASPTD
YVNVVNACLNVQSCVGITVFGVADPDSWRASTTPLLFDGNFNPKPAYNAIVQDLQQGSIEGRGHHHHHH-

>HG3.R3 (M49L, K50Q, T54I, Q90H, N102E, E131S, P154K, M172A, R190K, Q207M)
MAEAAQSVDQLIKARGKVYFGVATDQNRLTTGKNAAIIQADFGMVWPENSLQWDAIEPSQGNFNFAGADYLVNWAQQNGKLIG
GGMLVWHSHLPSWVSSITDKETLTNVMKNHITTLMTRYKGKIRAWDVVGSAFNEDGSLRQTVFLNVIGEDYIKIAFQTARAAD
PNAKLYIADYNLDSASYPKTQAIVNKVKQWRAAGVPIDGIGSMTHLSAGQGAGVLQALPLLASAGTPEVSILMLDVAGASPTD
YVNVVNACLNVQSCVGITVFGVADPDSWRASTTPLLFDGNFNPKPAYNAIVQDLQQGSIEGRGHHHHHH-

>HG3.R4 (I10M, M49L, K50Q, T54I, L69V, Q90H, N102E, A125C, E131S, P154K, M172A, R190K, Q207M, V266S)
MAEAAQSVDQLMKARGKVYFGVATDQNRLTTGKNAAIIQADFGMVWPENSLQWDAIEPSQGNFNFAGADYVVNWAQQNGKLIG
GGMLVWHSHLPSWVSSITDKETLTNVMKNHITTLMTRYKGKIRCWDVVGSAFNEDGSLRQTVFLNVIGEDYIKIAFQTARAAD
PNAKLYIADYNLDSASYPKTQAIVNKVKQWRAAGVPIDGIGSMTHLSAGQGAGVLQALPLLASAGTPEVSILMLDVAGASPTD
YVNVVNACLNVQSCVGITSFGVADPDSWRASTTPLLFDGNFNPKPAYNAIVQDLQQGSIEGRGHHHHHH-

>HG3.R5 (I10M, M49L, K50Q, T54V, L69V, Q90H, N102E, A125V, E131S, P154K, M172A, Y174S, R190K, Q207M, H209N, V266S)
MAEAAQSVDQLMKARGKVYFGVATDQNRLTTGKNAAIIQADFGMVWPENSLQWDAVEPSQGNFNFAGADYVVNWAQQNGKLIG
GGMLVWHSHLPSWVSSITDKETLTNVMKNHITTLMTRYKGKIRVWDVVGSAFNEDGSLRQTVFLNVIGEDYIKIAFQTARAAD
PNAKLYIADSNLDSASYPKTQAIVNKVKQWRAAGVPIDGIGSMTNLSAGQGAGVLQALPLLASAGTPEVSILMLDVAGASPTD
YVNVVNACLNVQSCVGITSFGVADPDSWRASTTPLLFDGNFNPKPAYNAIVQDLQQGSIEGRGHHHHHH-

>HG3.17 (V6I, Q37K, N47E, K50Q, G82A, M84C, S89N, Q90F, T105I, A125T, T208M, F267M, W275A, R276F, T279S, D300N)
MAEAAQSIDQLIKARGKVYFGVATDQNRLTTGKNAAIIKADFGMVWPEESMQWDATEPSQGNFNFAGADYLVNWAQQNGKLIG
AGCLVWHNFLPSWVSSITDKNTLINVMKNHITTLMTRYKGKIRTWDVVGEAFNEDGSLRQNVFLNVIGEDYIPIAFQTARAAD
PNAKLYIMDYNLDSASYPKTQAIVNRVKQWRAAGVPIDGIGSQMHLSAGQGAGVLQALPLLASAGTPEVSILMLDVAGASPTD
YVNVVNACLNVQSCVGITVMGVADPDSAFASSTPLLFDGNFNPKPAYNAIVQNLQQGSIEGRGHHHHHH-

>HG3.17wR5 (V6I, I10M, Q37K, N47E, M49L, K50Q, T54V, L69V, G82A, M84C, S89N, Q90F, N102E, T105I, A125T, E131S, P154K, M172A, Y174S, R190K, Q207M, T208M, H209N, V266S, F267M, W275A, R276F, T279S, D300N)
MAEAAQSIDQLMKARGKVYFGVATDQNRLTTGKNAAIIKADFGMVWPEESLQWDAVEPSQGNFNFAGADYVVNWAQQNGKLIG
AGCLVWHNFLPSWVSSITDKETLINVMKNHITTLMTRYKGKIRTWDVVGSAFNEDGSLRQNVFLNVIGEDYIKIAFQTARAAD
PNAKLYIADSNLDSASYPKTQAIVNKVKQWRAAGVPIDGIGSMMNLSAGQGAGVLQALPLLASAGTPEVSILMLDVAGASPTD
YVNVVNACLNVQSCVGITSMGVADPDSAFASSTPLLFDGNFNPKPAYNAIVQNLQQGSIEGRGHHHHHH-

>HG3.5w17 (V6I, I10M, Q37K, N47E, M49L, K50Q, T54V, L69V, G82A, M84C, S89N, Q90H, N102E, T105I, A125V, E131S, P154K, M172A, Y174S, R190K, Q207M, T208M, H209N, V266S, F267M, W275A, R276F, T279S, D300N)
MAEAAQSIDQLMKARGKVYFGVATDQNRLTTGKNAAIIKADFGMVWPEESLQWDAVEPSQGNFNFAGADYVVNWAQQNGKLIG
AGCLVWHNHLPSWVSSITDKETLINVMKNHITTLMTRYKGKIRVWDVVGSAFNEDGSLRQNVFLNVIGEDYIKIAFQTARAAD
PNAKLYIADSNLDSASYPKTQAIVNKVKQWRAAGVPIDGIGSMMNLSAGQGAGVLQALPLLASAGTPEVSILMLDVAGASPTD
YVNVVNACLNVQSCVGITSMGVADPDSAFASSTPLLFDGNFNPKPAYNAIVQNLQQGSIEGRGHHHHHH-

Nucleotide sequences of HG3 variants

>HG3

ATGGCAGAAGCAGCACAGAGCGTTGACCAGCTGATTAAAGCACGTGGTAAAGTTTATTTTGGTGTTGCCACCGATCAGAATCG
TCTGACCACCGGTAAAAATGCAGCAATTATTCAGGCAGATTTTGGTATGGTTTGGCCTGAAAATAGCATGAAATGGGATGCAA
CCGAACCGAGCCAGGGCAATTTTAACTTTGCCGGTGCAGATTATCTGGTTAATTGGGCACAGCAGAATGGTAAACTGATTGGT
GGTGGTATGCTGGTTTGGCATAGCCAGCTGCCGAGCTGGGTTAGCAGCATTACCGATAAAAACACCCTGACCAATGTGATGAA
AAACCATATCACCACACTGATGACCCGCTATAAAGGTAAAATTCGTGCATGGGATGTTGTGGGTGAAGCCTTTAATGAAGATG
GTAGCCTGCGTCAGACCGTTTTTCTGAATGTTATTGGCGAAGATTATATCCCGATTGCATTTCAGACCGCACGTGCAGCAGAT
CCGAATGCAAAACTGTATATCATGGATTATAACCTGGATAGCGCAAGCTATCCGAAAACACAGGCAATTGTTAATCGTGTTAA
ACAGTGGCGTGCAGCCGGTGTTCCGATTGATGGTATTGGTAGTCAGACCCATCTGAGCGCAGGTCAAGGTGCGGGTGTTCTGC
AGGCACTGCCGCTGCTGGCAAGCGCAGGTACACCGGAAGTTAGCATTCTGATGCTGGATGTTGCAGGCGCAAGCCCGACCGAT
TATGTTAATGTTGTTAATGCATGCCTGAATGTTCAGAGCTGTGTTGGTATTACCGTTTTTGGTGTGGCAGATCCGGATAGCTG
GCGTGCAAGCACCACACCGCTGCTGTTTGATGGTAATTTCAATCCGAAACCGGCATATAATGCCATTGTTCAGGATCTGCAGC
AGGGTAGCATTGAAGGTCGTGGTCATCATCACCATCATCATTAA

>HG3.R1

ATGGCAGAAGCAGCACAGAGCGTTGACCAGCTGATTAAAGCACGTGGTAAAGTTTATTTTGGTGTTGCCACCGATCAGAATCG
TCTGACCACCGGTAAAAATGCAGCAATTATTCAGGCAGATTTTGGTATGGTTTGGCCTGAAAATAGCATGCAGTGGGATGCAA
CCGAACCGAGCCAGGGCAATTTTAACTTTGCCGGTGCAGATTATCTGGTTAATTGGGCACAGCAGAATGGTAAACTGATTGGT
GGTGGTATGCTGGTTTGGCATAGCCAGCTGCCGAGCTGGGTTAGCAGCATTACCGATAAAAACACCCTGACCAATGTGATGAA
AAACCATATCACCACACTGATGACCCGCTATAAAGGTAAAATTCGTGCATGGGATGTTGTGGGTGAAGCCTTTAATGAAGATG
GTAGCCTGCGTCAGACCGTTTTTCTGAATGTTATTGGCGAAGATTATATCCCGATTGCATTTCAGACCGCACGTGCAGCAGAT
CCGAGCGCAAAACTGTATATCGCGGATTATAACCTGGATAGCGCAAGCTATCCGAAAACACAGGCAATTGTTAATCGTGTTAA
ACAGTGGCGTGCAGCCGGTGTTCCGATTGATGGTATTGGTAGTCAGACCCATCTGAGCGCAGGTCAAGGTGCGGGTGTTCTGC
AGGCACTGCCGCTGCTGGCAAGCGCAGGTACACCGGAAGTTAGCATTCTGATGCTGGATGTTGCAGGCGCAAGCCCGACCGAT
TATGTTAATGTTGTTAATGCATGCCTGAATGTTCAGAGCTGTGTTGGTATTACCGTTTTTGGTGTGGCAGATCCGGATAGCTG
GCGTGCAAGCACCACACCGCTGCTGTTTGATGGTAATTTCAATCCGAAACCGGCATATAATGCCATTGTTCAGGATCTGCAGC
AGGGTAGCATTGAAGGTCGTGGTCATCATCACCATCATCATTAA

>HG3.R2

ATGGCAGAAGCAGCACAGAGCGTTGACCAGCTGATTAAAGCACGTGGTAAAGTTTATTTTGGTGTTGCCACCGATCAGAATCG
TCTGACCACCGGTAAAAATGCAGCAATTATTCAGGCAGATTTTGGTATGGTTTGGCCTGAAAATAGCATGCAGTGGGATGCAA
CCGAACCGAGCCAGGGCAATTTTAACTTTGCCGGTGCAGATTATCTGGTTAATTGGGCACAGCAGAATGGTAAACTGATTGGT
GGTGGTATGCTGGTTTGGCATAGCCATCTGCCGAGCTGGGTTAGCAGCATTACCGATAAAAACACCCTGACCAATGTGATGAA
AAACCATATCACCACACTGATGACCCGCTATAAAGGTAAAATTCGTGCATGGGATGTTGTGGGTGAAGCCTTTAATGAAGATG
GTAGCCTGCGTCAGACCGTTTTTCTGAATGTTATTGGCGAAGATTATATCCCGATTGCATTTCAGACCGCACGTGCAGCAGAT
CCGAACGCAAAACTGTATATCGCGGATTATAACCTGGATAGCGCAAGCTATCCGAAAACACAGGCAATTGTTAATCGTGTTAA
ACAGTGGCGTGCAGCCGGTGTTCCGATTGATGGTATTGGTAGTATGACCCATCTGAGCGCAGGTCAAGGTGCGGGTGTTCTGC
AGGCACTGCCGCTGCTGGCAAGCGCAGGTACACCGGAAGTTAGCATTCTGATGCTGGATGTTGCAGGCGCAAGCCCGACCGAT
TATGTTAATGTTGTTAATGCATGCCTGAATGTTCAGAGCTGTGTTGGTATTACCGTTTTTGGTGTGGCAGATCCGGATAGCTG
GCGTGCAAGCACCACACCGCTGCTGTTTGATGGTAATTTCAATCCGAAACCGGCATATAATGCCATTGTTCAGGATCTGCAGC
AGGGTAGCATTGAAGGTCGTGGTCATCATCACCATCATCATTAA

>HG3.R3

ATGGCAGAAGCAGCACAGAGCGTTGACCAGCTGATTAAAGCACGTGGTAAAGTTTATTTTGGTGTTGCCACCGATCAGAATCG
TCTGACCACCGGTAAAAATGCAGCAATTATTCAGGCAGATTTTGGTATGGTTTGGCCTGAAAATAGCCTGCAGTGGGATGCAA
TTGAACCGAGCCAGGGCAATTTTAACTTTGCCGGTGCAGATTATCTGGTTAATTGGGCACAGCAGAATGGTAAACTGATTGGT
GGTGGTATGCTGGTTTGGCATAGCCATCTGCCGAGCTGGGTTAGCAGCATTACCGATAAAGAAACCCTGACCAATGTGATGAA
AAACCATATCACCACACTGATGACCCGCTATAAAGGTAAAATTCGTGCATGGGATGTTGTGGGTAGCGCCTTTAATGAAGATG
GTAGCCTGCGTCAGACCGTTTTTCTGAATGTTATTGGCGAAGATTATATCAAAATTGCATTTCAGACCGCACGTGCAGCAGAT
CCGAACGCAAAACTGTATATCGCGGATTATAACCTGGATAGCGCAAGCTATCCGAAAACACAGGCAATTGTTAATAAAGTTAA
ACAGTGGCGTGCAGCCGGTGTTCCGATTGATGGTATTGGTAGTATGACCCATCTGAGCGCAGGTCAAGGTGCGGGTGTTCTGC
AGGCACTGCCGCTGCTGGCAAGCGCAGGTACACCGGAAGTTAGCATTCTGATGCTGGATGTTGCAGGCGCAAGCCCGACCGAT
TATGTTAATGTTGTTAATGCATGCCTGAATGTTCAGAGCTGTGTTGGTATTACCGTTTTTGGTGTGGCAGATCCGGATAGCTG
GCGTGCAAGCACCACACCGCTGCTGTTTGATGGTAATTTCAATCCGAAACCGGCATATAATGCCATTGTTCAGGATCTGCAGC
AGGGTAGCATTGAAGGTCGTGGTCATCATCACCATCATCATTAA

>HG3.R4

ATGGCAGAAGCAGCACAGAGCGTTGACCAGCTGATGAAAGCACGTGGTAAAGTTTATTTTGGTGTGGCCACCGATCAGAATCG
TCTGACCACCGGTAAAAATGCAGCAATTATTCAGGCAGATTTTGGTATGGTTTGGCCTGAAAATAGCCTGCAGTGGGATGCAA
TTGAACCGAGCCAGGGCAATTTTAACTTTGCCGGTGCAGATTATGTGGTTAATTGGGCACAGCAGAATGGTAAACTGATTGGT
GGTGGTATGCTGGTTTGGCATAGCCATCTGCCGAGCTGGGTTAGCAGCATTACCGATAAAGAAACCCTGACCAATGTGATGAA
AAACCATATCACCACACTGATGACCCGCTATAAAGGTAAAATTCGTTGCTGGGATGTTGTGGGTAGCGCCTTTAATGAAGATG
GTAGCCTGCGTCAGACCGTTTTTCTGAATGTTATTGGCGAAGATTATATCAAAATTGCATTTCAGACCGCACGTGCAGCAGAT
CCGAACGCAAAACTGTATATCGCGGATTATAACCTGGATAGCGCAAGCTATCCGAAAACACAGGCAATTGTTAATAAAGTTAA
ACAGTGGCGTGCAGCCGGTGTTCCGATTGATGGTATTGGTAGTATGACCCATCTGAGCGCAGGTCAAGGTGCGGGTGTTCTGC
AGGCACTGCCGCTGCTGGCAAGCGCAGGTACACCGGAAGTTAGCATTCTGATGCTGGATGTTGCAGGCGCAAGCCCGACCGAT
TATGTTAATGTTGTTAATGCATGCCTGAATGTTCAGAGCTGTGTTGGTATTACCAGCTTTGGTGTGGCAGATCCGGATAGCTG
GCGTGCAAGCACCACACCGCTGCTGTTTGATGGTAATTTCAATCCGAAACCGGCATATAATGCCATTGTTCAGGATCTGCAGC
AGGGTAGCATTGAAGGTCGTGGTCATCATCACCATCATCATTAA

>HG3.R5

ATGGCAGAAGCAGCACAGAGCGTTGACCAGCTGATGAAAGCACGTGGTAAAGTTTATTTTGGTGTGGCCACCGATCAGAATCG
TCTGACCACCGGTAAAAATGCAGCAATTATTCAGGCAGATTTTGGTATGGTTTGGCCTGAAAATAGCCTGCAGTGGGATGCAG
TGGAACCGAGCCAGGGCAATTTTAACTTTGCCGGTGCAGATTATGTGGTTAATTGGGCACAGCAGAATGGTAAACTGATTGGT
GGTGGTATGCTGGTTTGGCATAGCCATCTGCCGAGCTGGGTTAGCAGCATTACCGATAAAGAAACCCTGACCAATGTGATGAA
AAACCATATCACCACACTGATGACCCGCTATAAAGGTAAAATTCGTGTGTGGGATGTTGTGGGTAGCGCCTTTAATGAAGATG
GTAGCCTGCGTCAGACCGTTTTTCTGAATGTTATTGGCGAAGATTATATCAAAATTGCATTTCAGACCGCACGTGCAGCAGAT
CCGAACGCAAAACTGTATATCGCGGATAGCAACCTGGATAGCGCAAGCTATCCGAAAACACAGGCAATTGTTAATAAAGTTAA
ACAGTGGCGTGCAGCCGGTGTTCCGATTGATGGTATTGGTAGTATGACCAACCTGAGCGCAGGTCAAGGTGCGGGTGTTCTGC
AGGCACTGCCGCTGCTGGCAAGCGCAGGTACACCGGAAGTTAGCATTCTGATGCTGGATGTTGCAGGCGCAAGCCCGACCGAT
TATGTTAATGTTGTTAATGCATGCCTGAATGTTCAGAGCTGTGTTGGTATTACCAGCTTTGGTGTGGCAGATCCGGATAGCTG
GCGTGCAAGCACCACACCGCTGCTGTTTGATGGTAATTTCAATCCGAAACCGGCATATAATGCCATTGTTCAGGATCTGCAGC
AGGGTAGCATTGAAGGTCGTGGTCATCATCACCATCATCATTAA

>HG3.17

ATGGCAGAAGCAGCACAGAGCATTGATCAACTGATTAAAGCACGTGGCAAAGTGTATTTTGGTGTTGCAACCGATCAGAATCG
TCTGACCACCGGTAAAAATGCAGCAATTATCAAAGCCGATTTTGGTATGGTTTGGCCTGAAGAAAGCATGCAGTGGGATGCAA
CCGAACCGAGCCAGGGCAATTTTAACTTTGCCGGTGCAGATTATCTGGTTAATTGGGCACAGCAGAATGGTAAACTGATTGGT
GCCGGTTGTCTGGTTTGGCATAATTTTCTGCCGAGCTGGGTTAGCAGCATTACCGATAAAAACACCCTGATCAACGTGATGAA
AAACCATATCACCACACTGATGACCCGCTATAAAGGTAAAATTCGTACCTGGGATGTTGTGGGTGAAGCCTTTAATGAAGATG
GTAGCCTGCGTCAGAATGTGTTTCTGAATGTTATTGGCGAAGATTATATCCCGATTGCATTTCAGACCGCACGTGCAGCAGAT
CCGAATGCAAAACTGTATATCATGGATTATAACCTGGATAGCGCAAGCTATCCGAAAACACAGGCAATTGTTAATCGTGTTAA
ACAGTGGCGTGCAGCCGGTGTTCCGATTGATGGTATTGGTAGCCAGATGCACCTGAGCGCAGGTCAAGGTGCGGGTGTTCTGC
AGGCACTGCCGCTGCTGGCAAGCGCAGGTACACCGGAAGTTAGCATTCTGATGCTGGATGTTGCAGGCGCAAGCCCGACCGAT
TATGTTAATGTTGTTAATGCATGCCTGAATGTTCAGAGCTGTGTTGGTATTACCGTTATGGGTGTTGCAGATCCGGATAGCGC
CTTTGCGAGCAGCACACCGCTGCTGTTTGATGGTAATTTCAATCCGAAACCGGCATATAATGCCATTGTTCAGAATCTGCAGC
AGGGTAGCATTGAAGGTCGTGGTCATCATCACCATCATCATTAA

>HG3.17wR5

ATGGCAGAAGCAGCACAGAGCATTGATCAACTGATGAAAGCACGTGGCAAAGTGTATTTTGGTGTTGCAACCGATCAGAATCG
TCTGACCACCGGTAAAAATGCAGCAATTATCAAAGCCGATTTTGGTATGGTTTGGCCTGAAGAAAGCCTGCAGTGGGATGCAG
TGGAACCGAGCCAGGGCAATTTTAACTTTGCCGGTGCAGATTATGTGGTTAATTGGGCACAGCAGAATGGTAAACTGATTGGT
GCCGGTTGTCTGGTTTGGCATAATTTTCTGCCGAGCTGGGTTAGCAGCATTACCGATAAAGAAACCCTGATCAACGTGATGAA
AAACCATATCACCACACTGATGACCCGCTATAAAGGTAAAATTCGTACCTGGGATGTTGTGGGTAGCGCCTTTAATGAAGATG
GTAGCCTGCGTCAGAATGTGTTTCTGAATGTTATTGGCGAAGATTATATCAAAATTGCATTTCAGACCGCACGTGCAGCAGAT
CCGAATGCAAAACTGTATATCGCGGATAGCAACCTGGATAGCGCAAGCTATCCGAAAACACAGGCAATTGTTAATAAAGTTAA
ACAGTGGCGTGCAGCCGGTGTTCCGATTGATGGTATTGGTAGCATGATGAATCTGAGCGCAGGTCAAGGTGCGGGTGTTCTGC
AGGCACTGCCGCTGCTGGCAAGCGCAGGTACACCGGAAGTTAGCATTCTGATGCTGGATGTTGCAGGCGCAAGCCCGACCGAT
TATGTTAATGTTGTTAATGCATGCCTGAATGTTCAGAGCTGTGTTGGTATTACCAGCATGGGTGTTGCAGATCCGGATAGCGC
CTTTGCGAGCAGCACACCGCTGCTGTTTGATGGTAATTTCAATCCGAAACCGGCATATAATGCCATTGTTCAGAATCTGCAGC
AGGGTAGCATTGAAGGTCGTGGTCATCATCACCATCATCATTA

>HG3.R5w17

ATGGCAGAAGCAGCACAGAGCATTGACCAGCTGATGAAAGCACGTGGTAAAGTTTATTTTGGTGTGGCCACCGATCAGAATCG
TCTGACCACCGGTAAAAATGCAGCAATTATTAAAGCAGATTTTGGTATGGTTTGGCCTGAAGAAAGCCTGCAGTGGGATGCAG
TGGAACCGAGCCAGGGCAATTTTAACTTTGCCGGTGCAGATTATGTGGTTAATTGGGCACAGCAGAATGGTAAACTGATTGGT
GCCGGTTGTCTGGTTTGGCATAATCATCTGCCGAGCTGGGTTAGCAGCATTACCGATAAAGAAACCCTGATCAATGTGATGAA
AAACCATATCACCACACTGATGACCCGCTATAAAGGTAAAATTCGTGTGTGGGATGTTGTGGGTAGCGCCTTTAATGAAGATG
GTAGCCTGCGTCAGAATGTTTTTCTGAATGTTATTGGCGAAGATTATATCAAAATTGCATTTCAGACCGCACGTGCAGCAGAT
CCGAACGCAAAACTGTATATCGCGGATAGCAACCTGGATAGCGCAAGCTATCCGAAAACACAGGCAATTGTTAATAAAGTTAA
ACAGTGGCGTGCAGCCGGTGTTCCGATTGATGGTATTGGTAGCATGATGAATCTGAGCGCAGGTCAAGGTGCGGGTGTTCTGC
AGGCACTGCCGCTGCTGGCAAGCGCAGGTACACCGGAAGTTAGCATTCTGATGCTGGATGTTGCAGGCGCAAGCCCGACCGAT
TATGTTAATGTTGTTAATGCATGCCTGAATGTTCAGAGCTGTGTTGGTATTACCAGCATGGGTGTGGCAGATCCGGATAGCGC
CTTTGCAAGCAGCACACCGCTGCTGTTTGATGGTAATTTCAATCCGAAACCGGCATATAATGCCATTGTTCAGAATCTGCAGC
AGGGTAGCATTGAAGGTCGTGGTCATCATCACCATCATCATTAA

Cartesian coordinates of cluster model QM structures calculated with PCM(H2O)/M06-2X/6-31G(d)

1-w_e (QM)

C	-2.27730000	-4.18000000	-1.83750000
H	-2.38340000	-3.63470000	-2.77790000
C	-0.84080000	-4.50710000	-1.49110000
C	-2.81230000	-3.34960000	-0.67690000
H	-2.83320000	-5.11860000	-1.91530000
N	0.15210000	-3.84040000	-2.11230000
O	-0.58630000	-5.26220000	-0.54910000
C	-4.26310000	-2.98620000	-0.78650000
H	-2.21890000	-2.42870000	-0.59390000
H	-2.63060000	-3.91330000	0.24530000
C	1.51630000	-3.94740000	-1.59610000
C	0.10210000	-3.10030000	-3.36370000
C	-5.34350000	-3.82430000	-0.70570000
C	-4.76420000	-1.66400000	-0.99150000
C	2.34400000	-3.17160000	-2.60440000
H	1.81070000	-4.99930000	-1.52800000
C	1.54160000	-3.29870000	-0.22610000
C	1.52810000	-3.26860000	-3.85360000
H	-0.14250000	-2.04510000	-3.18350000
H	-0.63460000	-3.52640000	-4.04580000
N	-6.49800000	-3.08820000	-0.81080000
H	-5.38320000	-4.89420000	-0.55380000
C	-6.16720000	-1.76730000	-0.97140000
C	-4.16490000	-0.40890000	-1.16370000
H	2.42050000	-2.12730000	-2.27870000
H	3.35420000	-3.57510000	-2.69890000
O	1.28430000	-2.10570000	-0.03860000
N	1.88200000	-4.11780000	0.78600000
H	1.80130000	-2.52410000	-4.60510000
H	1.63630000	-4.26030000	-4.30640000
H	-7.43600000	-3.45750000	-0.76700000
C	-6.99180000	-0.65480000	-1.11070000
H	-3.08060000	-0.30070000	-1.17210000
C	-4.98810000	0.68210000	-1.29660000
C	1.79320000	-3.67510000	2.16360000
H	1.86420000	-5.11280000	0.60200000
H	-8.07360000	-0.74890000	-1.08720000
C	-6.37640000	0.55010000	-1.27780000
H	-4.56370000	1.67460000	-1.43050000
C	2.74530000	-2.51270000	2.43260000
H	0.78450000	-3.32330000	2.39720000
H	2.04070000	-4.51760000	2.81220000
H	-6.96570000	1.45970000	-1.35180000
O	2.34600000	-1.45890000	2.91590000
N	4.03560000	-2.73660000	2.08440000
C	5.06270000	-1.74380000	2.30350000
H	4.30470000	-3.68880000	1.86710000
C	5.76180000	-1.24140000	1.04510000
H	4.60350000	-0.89020000	2.80030000
H	5.84740000	-2.13850000	2.95770000
O	6.41490000	-0.19820000	1.09940000
N	5.74830000	-2.01190000	-0.06140000
C	6.66510000	-1.73000000	-1.15470000
H	5.17090000	-2.84110000	-0.09450000
C	6.28900000	-0.53940000	-2.03040000
H	7.65650000	-1.50780000	-0.75250000
H	6.73790000	-2.61470000	-1.78900000
O	7.09160000	-0.18620000	-2.89040000
N	5.11600000	0.09000000	-1.80080000
C	4.81510000	1.38480000	-2.39350000
H	4.48720000	-0.22110000	-1.05580000
C	4.88110000	2.53950000	-1.40390000
H	5.53050000	1.56930000	-3.19700000
H	3.80830000	1.38760000	-2.82090000
O	4.52790000	3.65880000	-1.77710000
N	5.30900000	2.28330000	-0.16180000
H	5.66290000	1.36450000	0.09750000
C	5.36840000	3.37500000	0.78820000
H	4.45320000	3.96320000	0.68560000
C	5.50650000	2.86610000	2.21870000
H	6.20890000	4.03850000	0.54650000
H	6.43710000	2.29850000	2.32330000
C	4.36300000	1.97390000	2.65840000
H	5.58010000	3.73880000	2.87580000
H	4.36900000	1.05950000	2.05400000
C	2.97620000	2.53860000	2.58110000
H	4.52800000	1.65110000	3.69530000
N	1.99820000	1.61550000	2.61340000
O	2.74740000	3.75260000	2.50160000
H	1.04030000	1.90480000	2.45450000
H	2.21710000	0.62500000	2.53540000
H	0.12290000	-0.89640000	-0.85620000
O	-0.42550000	-0.12790000	-1.09650000
H	-0.03570000	0.61510000	-0.60190000
H	2.45370000	-0.66150000	0.27520000
O	3.12410000	0.04600000	0.24410000
H	2.62020000	0.87430000	0.12020000
H	1.58510000	4.10050000	1.04660000
N	0.89150000	4.33540000	0.33080000
C	0.61950000	3.36770000	-0.54760000
C	0.10640000	5.54030000	0.53220000
C	-0.49940000	3.59220000	-1.54320000
O	1.24700000	2.29120000	-0.55520000
H	-0.91600000	5.30000000	0.84670000
H	0.06550000	6.14570000	-0.37770000
H	0.58610000	6.12770000	1.31440000
N	-0.61730000	2.46020000	-2.41720000

H	-0.30590000	4.51390000	-2.11040000
H	-1.43140000	3.75030000	-0.99070000
C	-1.75030000	2.23830000	-3.11800000
H	0.07150000	1.72320000	-2.31920000
C	-1.70540000	1.02920000	-4.03560000
O	-2.71060000	3.00510000	-3.07430000
H	-0.97860000	0.29070000	-3.69330000
H	-1.43690000	1.36310000	-5.04200000
H	-2.69850000	0.57750000	-4.07500000
O	-0.84820000	1.42270000	1.66450000
N	-1.69790000	2.39300000	1.10150000
C	-1.61040000	0.44070000	2.17540000
C	-2.91570000	1.98500000	1.26590000
C	-2.95390000	0.72300000	1.95200000
C	-1.17840000	-0.69500000	2.85110000
H	-3.76820000	2.59960000	0.98510000
C	-3.94000000	-0.16270000	2.37390000
C	-2.14970000	-1.57450000	3.25530000
H	-0.12260000	-0.89950000	3.00880000
H	-4.99420000	0.01140000	2.19520000
C	-3.49330000	-1.30360000	3.03410000
H	-1.87950000	-2.49020000	3.76760000
N	-4.48790000	-2.28930000	3.45860000
O	-5.65370000	-2.08940000	3.16240000
O	-4.09890000	-3.26170000	4.08340000
O	-5.42390000	3.53360000	1.34360000
C	-5.45930000	4.04170000	0.18590000
C	-4.31360000	4.94240000	-0.18720000
O	-6.34160000	3.83500000	-0.68200000
C	-4.63890000	5.84720000	-1.37320000
H	-3.45990000	4.29820000	-0.45050000
H	-4.00230000	5.51770000	0.69130000
H	-5.49080000	6.49470000	-1.14460000
H	-3.78810000	6.48340000	-1.64010000
H	-4.90830000	5.24080000	-2.23960000

1-w_e TS (QM)

C	-2.26060000	-4.18660000	-1.84150000
H	-2.36700000	-3.64150000	-2.78200000
C	-0.82360000	-4.51010000	-1.49390000
C	-2.79880000	-3.35740000	-0.68150000
H	-2.81410000	-5.12670000	-1.92010000
N	0.16820000	-3.84120000	-2.11430000
O	-0.56820000	-5.26490000	-0.55180000
C	-4.25040000	-2.99760000	-0.79250000
H	-2.20770000	-2.43490000	-0.59740000
H	-2.61730000	-3.92100000	0.24080000
C	1.53220000	-3.94460000	-1.59670000
C	0.11750000	-3.10120000	-3.36570000
C	-5.32870000	-3.83840000	-0.71260000
C	-4.75450000	-1.67670000	-0.99810000
C	2.35890000	-3.16690000	-2.60440000
H	1.82920000	-4.99580000	-1.52860000
C	1.55450000	-3.29570000	-0.22680000
C	1.54430000	-3.26600000	-3.85430000
H	-0.13080000	-2.04690000	-3.18640000
H	-0.61750000	-3.52930000	-4.04850000
N	-6.48510000	-3.10520000	-0.81880000
H	-5.36600000	-4.90830000	-0.56060000
C	-6.15730000	-1.78350000	-0.97920000
C	-4.15820000	-0.42010000	-1.16990000
H	2.43290000	-2.12250000	-2.27860000
H	3.36990000	-3.56840000	-2.69820000
O	1.29290000	-2.10350000	-0.04120000
N	1.89650000	-4.11390000	0.78580000
H	1.81640000	-2.52100000	-4.60570000
H	1.65510000	-4.25750000	-4.30690000
H	-7.42230000	-3.47680000	-0.77530000
C	-6.98460000	-0.67300000	-1.11950000
H	-3.07460000	-0.30740000	-1.17390000
C	-4.98400000	0.66880000	-1.30370000
C	1.80500000	-3.67120000	2.16320000
H	1.88060000	-5.10890000	0.60180000
H	-8.06610000	-0.76920000	-1.09980000
C	-6.37190000	0.53340000	-1.28610000
H	-4.54770000	1.65550000	-1.43940000
C	2.75390000	-2.50640000	2.43280000
H	0.79520000	-3.32080000	2.39560000
H	2.05400000	-4.51270000	2.81250000
H	-6.97660000	1.43190000	-1.38060000
O	2.35500000	-1.45560000	2.92120000
N	4.04520000	-2.72690000	2.08310000
C	5.06960000	-1.73170000	2.30570000
H	4.31680000	-3.67800000	1.86440000
C	5.76850000	-1.22780000	1.04800000
H	4.60680000	-0.87960000	2.80180000
H	5.85440000	-2.12460000	2.96110000
O	6.41950000	-0.18330000	1.10240000
N	5.75780000	-1.99840000	-0.05850000
C	6.67500000	-1.71440000	-1.15100000
H	5.18150000	-2.82820000	-0.09240000
C	6.29680000	-0.52490000	-2.02710000
H	7.66550000	-1.48960000	-0.74770000
H	6.75070000	-2.59900000	-1.78510000
O	7.09950000	-0.16990000	-2.88650000
N	5.12200000	0.10170000	-1.79890000
C	4.81840000	1.39560000	-2.39180000
H	4.49210000	-0.20940000	-1.05470000
C	4.88070000	2.55060000	-1.40230000
H	5.53380000	1.58230000	-3.19480000
H	3.81180000	1.39580000	-2.81960000
O	4.52710000	3.66950000	-1.77690000
N	5.30900000	2.29610000	-0.16030000
H	5.65940000	1.37660000	0.10130000
C	5.36390000	3.38760000	0.79020000
H	4.44680000	3.97280000	0.68730000
C	5.50200000	2.87920000	2.22090000
H	6.20230000	4.05390000	0.54920000
H	6.43440000	2.31460000	2.32640000
C	4.36030000	1.98420000	2.65960000
H	5.57240000	3.75240000	2.87760000
H	4.36960000	1.07050000	2.05430000
C	2.97220000	2.54550000	2.58110000
H	4.52670000	1.66120000	3.69630000
N	1.99800000	1.61830000	2.60120000
O	2.74310000	3.76050000	2.50970000
H	1.03630000	1.88390000	2.40880000
H	2.23150000	0.63250000	2.50940000
H	0.08030000	-0.88570000	-0.79700000
O	-0.50260000	-0.14270000	-1.03670000
H	-0.29990000	0.54920000	-0.38100000
H	2.45460000	-0.65130000	0.25640000
O	3.11860000	0.06220000	0.23120000
H	2.60520000	0.88490000	0.10400000
H	1.57730000	4.10580000	1.04580000
N	0.88740000	4.34100000	0.32730000
C	0.61650000	3.36840000	-0.55020000
C	0.09670000	5.53980000	0.52920000
C	-0.50230000	3.59000000	-1.54630000
O	1.25660000	2.30350000	-0.56570000
H	-0.90790000	5.29730000	0.89590000
H	0.00690000	6.11640000	-0.39610000
H	0.60270000	6.15910000	1.26940000
N	-0.63750000	2.42870000	-2.38160000
H	-0.28860000	4.48490000	-2.14790000
H	-1.42700000	3.78460000	-0.99420000
C	-1.74840000	2.23280000	-3.12240000
H	-0.00590000	1.65460000	-2.19750000
C	-1.69970000	1.02360000	-4.03970000
O	-2.68370000	3.03290000	-3.13250000

H	-1.01520000	0.26080000	-3.66460000
H	-1.36530000	1.35300000	-5.02770000
H	-2.70310000	0.60480000	-4.13580000
O	-0.79230000	1.37200000	1.66730000
N	-1.82730000	2.47580000	1.06440000
C	-1.58090000	0.44810000	2.16170000
C	-2.98000000	2.00850000	1.27690000
C	-2.95020000	0.72080000	1.95450000
C	-1.17460000	-0.69840000	2.84770000
H	-4.25020000	2.76700000	1.15010000
C	-3.93710000	-0.17300000	2.36790000
C	-2.14410000	-1.58020000	3.25100000
H	-0.12040000	-0.90760000	3.00980000
H	-4.99050000	0.00410000	2.18700000
C	-3.48820000	-1.31260000	3.02860000
H	-1.87450000	-2.49580000	3.76450000
N	-4.47390000	-2.30010000	3.44420000
O	-5.64540000	-2.10460000	3.15480000
O	-4.08810000	-3.28220000	4.06130000
O	-5.28210000	3.14060000	1.12270000
C	-5.46490000	4.02740000	0.17800000
C	-4.32110000	4.93090000	-0.19430000
O	-6.56040000	4.16760000	-0.35430000
C	-4.64750000	5.83480000	-1.38060000
H	-3.44840000	4.30050000	-0.41430000
H	-4.05000000	5.51230000	0.69570000
H	-5.50770000	6.46980000	-1.15850000
H	-3.79300000	6.47280000	-1.62240000
H	-4.89040000	5.23420000	-2.26030000

1 (QM)

C	-2.27920000	-4.18940000	-1.85360000
H	-2.38500000	-3.64680000	-2.79560000
C	-0.84300000	-4.51520000	-1.50480000
C	-2.81600000	-3.35670000	-0.69540000
H	-2.83550000	-5.12800000	-1.92900000
N	0.15080000	-3.85790000	-2.13140000
O	-0.59200000	-5.26410000	-0.55600000
C	-4.26680000	-2.99410000	-0.80760000
H	-2.22210000	-2.43710000	-0.61190000
H	-2.63510000	-3.91810000	0.22810000
C	1.51420000	-3.95480000	-1.60800000
C	0.10160000	-3.11200000	-3.37920000
C	-5.34690000	-3.83250000	-0.72630000
C	-4.76810000	-1.67260000	-1.01610000
C	2.34260000	-3.18070000	-2.61700000
H	1.81240000	-5.00610000	-1.54020000
C	1.53710000	-3.30310000	-0.23950000
C	1.52830000	-3.28080000	-3.86700000
H	-0.14300000	-2.05780000	-3.19770000
H	-0.63440000	-3.53520000	-4.06400000
N	-6.50120000	-3.09700000	-0.83460000
H	-5.38630000	-4.90200000	-0.57210000
C	-6.17110000	-1.77640000	-0.99740000
C	-4.16910000	-0.41760000	-1.19030000
H	2.41950000	-2.13680000	-2.29210000
H	3.35240000	-3.58570000	-2.71090000
O	1.28310000	-2.11250000	-0.07100000
N	1.88190000	-4.12650000	0.77800000
H	1.80170000	-2.53830000	-4.62070000
H	1.63620000	-4.27360000	-4.31750000
H	-7.43930000	-3.46640000	-0.79110000
C	-6.99600000	-0.66450000	-1.14020000
H	-3.08640000	-0.30890000	-1.19690000
C	-4.99270000	0.67280000	-1.32660000
C	1.78620000	-3.67420000	2.15150000
H	1.81300000	-5.12090000	0.60290000
H	-8.07780000	-0.75900000	-1.11720000
C	-6.38090000	0.54030000	-1.30920000
H	-4.56970000	1.66590000	-1.45960000
C	2.73740000	-2.51080000	2.41900000
H	0.77760000	-3.31950000	2.38320000
H	2.03260000	-4.51110000	2.80820000
H	-6.96890000	1.45050000	-1.38310000
O	2.34060000	-1.46000000	2.91150000
N	4.02770000	-2.73320000	2.06940000
C	5.05480000	-1.74110000	2.29090000
H	4.29340000	-3.68320000	1.83910000
C	5.75520000	-1.24120000	1.03250000
H	4.59370000	-0.88720000	2.78530000
H	5.83860000	-2.13490000	2.94700000
O	6.40860000	-0.19790000	1.08480000
N	5.74380000	-2.01430000	-0.07230000
C	6.66130000	-1.73420000	-1.16520000
H	5.16220000	-2.84040000	-0.10680000
C	6.28590000	-0.54580000	-2.04400000
H	7.65220000	-1.51060000	-0.76230000
H	6.73540000	-2.62020000	-1.79760000
O	7.08930000	-0.19410000	-2.90400000
N	5.11210000	0.08360000	-1.81720000
C	4.81160000	1.37710000	-2.41300000
H	4.48120000	-0.22460000	-1.07190000
C	4.87590000	2.53400000	-1.42590000
H	5.52820000	1.56070000	-3.21560000
H	3.80540000	1.37860000	-2.84200000
O	4.53170000	3.65420000	-1.80460000
N	5.29790000	2.27980000	-0.18150000
H	5.64760000	1.36060000	0.08170000
C	5.36020000	3.37450000	0.76500000
H	4.44640000	3.96470000	0.66090000
C	5.49680000	2.86880000	2.19680000
H	6.20220000	4.03520000	0.52120000
H	6.42760000	2.30180000	2.30350000
C	4.35310000	1.97700000	2.63700000
H	5.56920000	3.74280000	2.85220000
H	4.36060000	1.06070000	2.03520000
C	2.96630000	2.54090000	2.55680000
H	4.51680000	1.65730000	3.67510000
N	1.98890000	1.61730000	2.58160000
O	2.73850000	3.75580000	2.48710000
H	1.03210000	1.90830000	2.41910000
H	2.20980000	0.62900000	2.48210000
H	2.42690000	-0.66060000	0.20820000
O	3.10450000	0.04240000	0.21390000
H	2.60860000	0.87310000	0.07260000
H	1.57760000	4.10190000	1.01890000
N	0.87050000	4.32610000	0.31460000
C	0.61340000	3.36250000	-0.57700000
C	0.09750000	5.53700000	0.49790000
C	-0.50490000	3.58400000	-1.57370000
O	1.27690000	2.31520000	-0.62220000
H	-0.93180000	5.31320000	0.80310000
H	0.07280000	6.13830000	-0.41590000
H	0.57290000	6.12620000	1.28170000
N	-0.64600000	2.42450000	-2.40390000
H	-0.29280000	4.48240000	-2.17150000
H	-1.43520000	3.77600000	-1.02980000
C	-1.75330000	2.22630000	-3.14750000
H	0.11870000	1.76150000	-2.38240000
C	-1.70670000	1.01520000	-4.06230000
O	-2.71440000	2.99180000	-3.11550000
H	-0.78600000	0.43910000	-3.95970000
H	-1.79990000	1.36020000	-5.09510000
H	-2.56500000	0.37320000	-3.84570000

O	-0.86660000	1.46220000	1.70330000
N	-1.71840000	2.44280000	1.16380000
C	-1.62290000	0.46010000	2.18310000
C	-2.93410000	2.01990000	1.30500000
C	-2.96660000	0.73790000	1.95330000
C	-1.18740000	-0.69380000	2.82880000
H	-3.78900000	2.63810000	1.03640000
C	-3.94870000	-0.16370000	2.34700000
C	-2.15890000	-1.57270000	3.23370000
H	-0.13130000	-0.89870000	2.98400000
H	-5.00250000	0.00460000	2.16030000
C	-3.50230000	-1.30280000	3.01030000
H	-1.88810000	-2.49250000	3.73840000
N	-4.49580000	-2.29320000	3.42630000
O	-5.66100000	-2.09620000	3.12540000
O	-4.10700000	-3.26570000	4.05110000
O	-5.45260000	3.55680000	1.31870000
C	-5.46700000	4.03540000	0.14810000
C	-4.32130000	4.93580000	-0.22580000
O	-6.32950000	3.80030000	-0.73250000
C	-4.64540000	5.83780000	-1.41400000
H	-3.46820000	4.28980000	-0.48680000
H	-4.01060000	5.51310000	0.65160000
H	-5.49470000	6.48930000	-1.18610000
H	-3.79310000	6.47050000	-1.68410000
H	-4.91840000	5.22930000	-2.27770000

1 TS (QM)

C	-2.26120000	-4.20180000	-1.85170000
H	-2.36720000	-3.66150000	-2.79500000
C	-0.82450000	-4.52270000	-1.50020000
C	-2.80200000	-3.36830000	-0.69600000
H	-2.81460000	-5.14220000	-1.92600000
N	0.16820000	-3.86390000	-2.12690000
O	-0.57270000	-5.26930000	-0.54980000
C	-4.25370000	-3.01010000	-0.81090000
H	-2.21080000	-2.44690000	-0.61270000
H	-2.62120000	-3.92730000	0.22900000
C	1.53100000	-3.95570000	-1.60140000
C	0.11860000	-3.12080000	-3.37630000
C	-5.33150000	-3.85150000	-0.72930000
C	-4.75850000	-1.69060000	-1.02290000
C	2.35880000	-3.18140000	-2.61090000
H	1.83210000	-5.00600000	-1.53110000
C	1.55050000	-3.30100000	-0.23420000
C	1.54640000	-3.28650000	-3.86180000
H	-0.12930000	-2.06690000	-3.19750000
H	-0.61520000	-3.54760000	-4.06130000
N	-6.48790000	-3.11960000	-0.84100000
H	-5.36810000	-4.92080000	-0.57310000
C	-6.16130000	-1.79840000	-1.00590000
C	-4.16300000	-0.43420000	-1.19890000
H	2.43220000	-2.13670000	-2.28790000
H	3.36980000	-3.58390000	-2.70250000
O	1.29300000	-2.11100000	-0.06870000
N	1.89650000	-4.12170000	0.78550000
H	1.81860000	-2.54460000	-4.61660000
H	1.65800000	-4.27980000	-4.31010000
H	-7.42490000	-3.49160000	-0.79730000
C	-6.98920000	-0.68920000	-1.15220000
H	-3.08130000	-0.31980000	-1.20060000
C	-4.98940000	0.65350000	-1.33860000
C	1.79720000	-3.66650000	2.15780000
H	1.82800000	-5.11650000	0.61240000
H	-8.07060000	-0.78620000	-1.13390000
C	-6.37730000	0.51700000	-1.32280000
H	-4.55380000	1.64010000	-1.47890000
C	2.74470000	-2.49970000	2.42420000
H	0.78720000	-3.31310000	2.38700000
H	2.04520000	-4.50090000	2.81700000
H	-6.98130000	1.41510000	-1.42230000
O	2.34760000	-1.45120000	2.91960000
N	4.03610000	-2.71880000	2.07400000
C	5.06000000	-1.72370000	2.29780000
H	4.30480000	-3.66790000	1.84380000
C	5.76060000	-1.22430000	1.03920000
H	4.59510000	-0.87030000	2.78960000
H	5.84370000	-2.11420000	2.95590000
O	6.41190000	-0.17960000	1.08990000
N	5.75290000	-1.99980000	-0.06410000
C	6.67120000	-1.71930000	-1.15630000
H	5.17230000	-2.82650000	-0.09840000
C	6.29350000	-0.53380000	-2.03800000
H	7.66080000	-1.49180000	-0.75230000
H	6.74900000	-2.60650000	-1.78660000
O	7.09740000	-0.18150000	-2.89740000
N	5.11760000	0.09250000	-1.81440000
C	4.81420000	1.38400000	-2.41310000
H	4.48560000	-0.21600000	-1.07020000
C	4.87380000	2.54310000	-1.42830000
H	5.53140000	1.56820000	-3.21510000
H	3.80870000	1.38160000	-2.84350000
O	4.52840000	3.66190000	-1.81050000
N	5.29480000	2.29290000	-0.18300000
H	5.64340000	1.37430000	0.08360000
C	5.35270000	3.38960000	0.76140000
H	4.43710000	3.97670000	0.65560000
C	5.48870000	2.88730000	2.19440000
H	6.19290000	4.05230000	0.51700000
H	6.42160000	2.32400000	2.30370000
C	4.34710000	1.99310000	2.63490000
H	5.55730000	3.76320000	2.84780000
H	4.35840000	1.07620000	2.03450000
C	2.95860000	2.55290000	2.55180000
H	4.51190000	1.67560000	3.67360000
N	1.98770000	1.62400000	2.57070000
O	2.72880000	3.76830000	2.48050000
H	1.02130000	1.88470000	2.39270000
H	2.22400000	0.63830000	2.48280000
H	2.42990000	-0.65240000	0.21020000
O	3.10730000	0.05070000	0.20820000
H	2.60880000	0.88180000	0.07730000
H	1.56000000	4.10050000	1.01550000
N	0.85680000	4.32420000	0.30700000
C	0.60740000	3.36070000	-0.58680000
C	0.08420000	5.53630000	0.48260000
C	-0.50960000	3.57750000	-1.58590000
O	1.28210000	2.32090000	-0.63820000
H	-0.94940000	5.31140000	0.77200000
H	0.07470000	6.14150000	-0.42920000
H	0.54910000	6.12150000	1.27580000
N	-0.63320000	2.43190000	-2.43780000
H	-0.31310000	4.49030000	-2.16680000
H	-1.44230000	3.74010000	-1.03640000
C	-1.75210000	2.21260000	-3.15830000
H	0.13610000	1.77400000	-2.41910000
C	-1.70080000	0.99970000	-4.07060000
O	-2.72850000	2.95800000	-3.10460000
H	-0.76320000	0.44770000	-3.99200000
H	-1.83290000	1.33680000	-5.10170000
H	-2.53590000	0.33720000	-3.82640000

O	-0.81410000	1.44200000	1.76190000
N	-1.87240000	2.56610000	1.17400000
C	-1.59340000	0.48640000	2.18790000
C	-3.01000000	2.05630000	1.33950000
C	-2.96780000	0.74330000	1.96370000
C	-1.18600000	-0.69320000	2.82480000
H	-4.29700000	2.82060000	1.19600000
C	-3.94810000	-0.17220000	2.33820000
C	-2.15540000	-1.57420000	3.23020000
H	-0.13170000	-0.90270000	2.98550000
H	-5.00080000	-0.00410000	2.14500000
C	-3.49930000	-1.30870000	3.00440000
H	-1.88460000	-2.49580000	3.73240000
N	-4.48240000	-2.30110000	3.40870000
O	-5.65370000	-2.11090000	3.11250000
O	-4.09740000	-3.28340000	4.02730000
O	-5.31900000	3.18020000	1.12420000
C	-5.47560000	4.01790000	0.12840000
C	-4.33200000	4.92070000	-0.24580000
O	-6.55200000	4.11250000	-0.44930000
C	-4.65710000	5.81940000	-1.43640000
H	-3.46060000	4.28690000	-0.46240000
H	-4.05960000	5.50470000	0.64190000
H	-5.51800000	6.45510000	-1.21850000
H	-3.80230000	6.45690000	-1.67830000
H	-4.89790000	5.21510000	-2.31400000

1-Q50A-w_e (QM)

C	-1.98450000	-4.00800000	-2.06010000
H	-2.08930000	-3.40100000	-2.96190000
C	-0.54510000	-4.30900000	-1.70190000
C	-2.57530000	-3.28150000	-0.85760000
H	-2.50390000	-4.95760000	-2.21760000
N	0.43630000	-3.56480000	-2.24880000
O	-0.28490000	-5.12160000	-0.81080000
C	-4.03550000	-2.96170000	-0.97760000
H	-2.01710000	-2.34900000	-0.69650000
H	-2.39370000	-3.90250000	0.02710000
C	1.79170000	-3.66190000	-1.70820000
C	0.38780000	-2.74050000	-3.44660000
C	-5.08620000	-3.84040000	-0.98300000
C	-4.57950000	-1.64650000	-1.10150000
C	2.61320000	-2.78800000	-2.63840000
H	2.12240000	-4.70530000	-1.70690000
C	1.76260000	-3.11190000	-0.29580000
C	1.82960000	-2.82400000	-3.91130000
H	0.09930000	-1.71000000	-3.20080000
H	-0.31630000	-3.14390000	-4.17560000
N	-6.26410000	-3.13920000	-1.06490000
H	-5.09030000	-4.91910000	-0.90770000
C	-5.97800000	-1.79950000	-1.12370000
C	-4.02260000	-0.36220000	-1.16990000
H	2.64400000	-1.76750000	-2.23780000
H	3.63920000	-3.14840000	-2.73550000
O	1.45380000	-1.94690000	-0.03090000
N	2.10990000	-3.99010000	0.66390000
H	2.09200000	-2.01870000	-4.60160000
H	1.98390000	-3.77650000	-4.43010000
H	-7.18880000	-3.54310000	-1.06990000
C	-6.83920000	-0.70920000	-1.20500000
H	-2.94320000	-0.21660000	-1.14110000
C	-4.88180000	0.70620000	-1.24620000
C	1.97410000	-3.64870000	2.06710000
H	2.13190000	-4.96920000	0.40860000
H	-7.91710000	-0.84220000	-1.21580000
C	-6.26440000	0.52520000	-1.27140000
H	-4.49200000	1.72040000	-1.29550000
C	2.87690000	-2.47610000	2.44090000
H	0.94810000	-3.34850000	2.29830000
H	2.23690000	-4.52510000	2.66240000
H	-6.88390000	1.41730000	-1.29620000
O	2.43700000	-1.49100000	3.01490000
N	4.18270000	-2.62610000	2.09590000
C	5.16730000	-1.61990000	2.42400000
H	4.48930000	-3.54780000	1.80910000
C	5.87570000	-1.00530000	1.22220000
H	4.66160000	-0.81730000	2.95990000
H	5.94830000	-2.03260000	3.07180000
O	6.49280000	0.05140000	1.36240000
N	5.91460000	-1.69550000	0.06280000
C	6.84530000	-1.30430000	-0.98340000
H	5.36900000	-2.53950000	-0.04360000
C	6.44600000	-0.06820000	-1.78220000
H	7.81750000	-1.07440000	-0.54050000
H	6.96760000	-2.13820000	-1.67630000
O	7.25780000	0.37550000	-2.59100000
N	5.24590000	0.50130000	-1.54180000
C	4.91160000	1.82480000	-2.04570000
H	4.60410000	0.11070000	-0.84680000
C	4.91330000	2.90760000	-0.97590000
H	5.63690000	2.09300000	-2.81620000
H	3.91490000	1.82340000	-2.49620000
O	4.52000000	4.03560000	-1.27800000
N	5.33880000	2.58450000	0.25060000
H	5.69010000	1.65310000	0.46520000
C	5.32060000	3.60130000	1.28100000
H	4.37870000	4.15410000	1.18800000
C	5.44490000	2.99690000	2.67520000
H	6.12760000	4.32480000	1.10800000
H	6.40630000	2.47240000	2.74430000
C	4.32510000	2.03640000	3.02220000
H	5.48350000	3.82070000	3.39610000
H	4.24110000	1.23890000	2.27910000
H	4.48850000	1.58040000	4.00390000
H	3.35960000	2.55640000	3.05430000
H	0.31890000	-0.72780000	-0.84000000
O	-0.24560000	0.03580000	-1.06010000
H	0.06160000	0.74330000	-0.46630000
H	2.55830000	-0.48320000	0.48070000
O	3.19840000	0.24300000	0.37630000
H	2.66920000	1.06190000	0.35260000
O	1.27240000	2.47810000	-0.23370000
C	0.60650000	3.52440000	-0.17050000
N	0.83830000	4.45280000	0.76410000
C	-0.49730000	3.78050000	-1.17490000
H	1.53210000	4.18860000	1.45270000
C	-0.00970000	5.59560000	1.04680000
N	-0.55690000	2.70670000	-2.12450000
H	-0.31840000	4.74280000	-1.67530000
H	-1.44720000	3.87260000	-0.63860000
H	-0.98680000	5.27920000	1.42890000
H	-0.15600000	6.20090000	0.14860000
H	0.48350000	6.21250000	1.79640000
C	-1.66260000	2.49940000	-2.87200000
H	0.15920000	1.99150000	-2.07070000
C	-1.55310000	1.36120000	-3.87120000
O	-2.65040000	3.22790000	-2.80100000
H	-0.76100000	0.66030000	-3.60420000
H	-1.34930000	1.78400000	-4.85910000
H	-2.50950000	0.83580000	-3.91380000
H	-3.50540000	4.30400000	-0.11030000

C	-4.38770000	4.89740000	0.17670000
C	-5.50800000	3.93340000	0.45700000
C	-4.71890000	5.87270000	-0.95020000
H	-4.11670000	5.41920000	1.10080000
O	-5.48450000	3.35290000	1.58000000
O	-6.35590000	3.75060000	-0.45050000
H	-5.59470000	6.47680000	-0.69430000
H	-3.88400000	6.55160000	-1.15490000
H	-4.95450000	5.32140000	-1.86150000
H	-3.77160000	2.49890000	1.18490000
C	-2.90840000	1.89260000	1.44760000
N	-1.69870000	2.34510000	1.34110000
C	-2.92230000	0.58670000	2.04640000
O	-0.83100000	1.37060000	1.85890000
C	-1.57330000	0.33610000	2.28230000
C	-3.88640000	-0.36120000	2.38070000
C	-1.11850000	-0.82990000	2.88820000
H	-4.94180000	-0.21140000	2.18790000
C	-3.41340000	-1.52980000	2.97000000
C	-2.06610000	-1.76900000	3.20510000
H	-0.05940000	-1.00650000	3.05630000
N	-4.38090000	-2.57640000	3.30000000
H	-1.77420000	-2.70880000	3.65860000
O	-5.54790000	-2.39390000	2.99630000
O	-3.97030000	-3.57990000	3.85840000

1-Q50A-w_e TS (QM)

C	-1.96700000	-4.01120000	-2.06680000
H	-2.07230000	-3.40350000	-2.96830000
C	-0.52730000	-4.30900000	-1.70760000
C	-2.56040000	-3.28680000	-0.86440000
H	-2.48390000	-4.96190000	-2.22600000
N	0.45300000	-3.56300000	-2.25400000
O	-0.26580000	-5.12170000	-0.81690000
C	-4.02130000	-2.97030000	-0.98520000
H	-2.00510000	-2.35240000	-0.70290000
H	-2.37850000	-3.90790000	0.02020000
C	1.80820000	-3.65650000	-1.71180000
C	0.40340000	-2.73730000	-3.45070000
C	-5.07000000	-3.85140000	-0.99200000
C	-4.56830000	-1.65630000	-1.10870000
C	2.62820000	-2.78000000	-2.64080000
H	2.14150000	-4.69900000	-1.71120000
C	1.77650000	-3.10740000	-0.29900000
C	1.84570000	-2.81710000	-3.91430000
H	0.11270000	-1.70770000	-3.20390000
H	-0.29970000	-3.14060000	-4.18060000
N	-6.24960000	-3.15290000	-1.07450000
H	-5.07180000	-4.93020000	-0.91750000
C	-5.96640000	-1.81250000	-1.13210000
C	-4.01430000	-0.37070000	-1.17580000
H	2.65740000	-1.76010000	-2.23920000
H	3.65490000	-3.13880000	-2.73790000
O	1.46320000	-1.94330000	-0.03590000
N	2.12540000	-3.98540000	0.66040000
H	2.10690000	-2.01100000	-4.60410000
H	2.00230000	-3.76910000	-4.43340000
H	-7.17340000	-3.55910000	-1.08000000
C	-6.83010000	-0.72410000	-1.21340000
H	-2.93570000	-0.22110000	-1.13910000
C	-4.87590000	0.69580000	-1.25210000
C	1.98750000	-3.64530000	2.06370000
H	2.14990000	-4.96400000	0.40410000
H	-7.90760000	-0.85860000	-1.22770000
C	-6.25800000	0.51160000	-1.27850000
H	-4.47400000	1.70500000	-1.30040000
C	2.88730000	-2.47080000	2.43890000
H	0.96060000	-3.34660000	2.29410000
H	2.25210000	-4.52110000	2.65900000
H	-6.89340000	1.39250000	-1.32390000
O	2.44850000	-1.49170000	3.02250000
N	4.19350000	-2.61630000	2.09100000
C	5.17570000	-1.60940000	2.42450000
H	4.50220000	-3.53620000	1.80060000
C	5.88360000	-0.99230000	1.22350000
H	4.66740000	-0.80860000	2.96050000
H	5.95670000	-2.02170000	3.07250000
O	6.49840000	0.06570000	1.36450000
N	5.92480000	-1.68160000	0.06360000
C	6.85560000	-1.28770000	-0.98150000
H	5.37990000	-2.52570000	-0.04450000
C	6.45410000	-0.05200000	-1.77980000
H	7.82680000	-1.05590000	-0.53750000
H	6.98040000	-2.12080000	-1.67480000
O	7.26530000	0.39360000	-2.58830000
N	5.25270000	0.51490000	-1.53950000
C	4.91550000	1.83760000	-2.04330000
H	4.61010000	0.12520000	-0.84450000
C	4.91390000	2.91980000	-0.97280000
H	5.64040000	2.10820000	-2.81330000
H	3.91880000	1.83390000	-2.49390000
O	4.51960000	4.04750000	-1.27490000
N	5.34130000	2.59770000	0.25320000
H	5.68900000	1.66530000	0.46900000
C	5.31780000	3.61290000	1.28490000
H	4.37390000	4.16250000	1.19130000
C	5.44240000	3.00790000	2.67880000
H	6.12230000	4.33970000	1.11390000
H	6.40500000	2.48560000	2.74780000
C	4.32450000	2.04460000	3.02430000
H	5.47900000	3.83120000	3.40040000
H	4.23560000	1.25320000	2.27500000
H	4.49310000	1.58100000	4.00160000
H	3.35930000	2.56430000	3.06550000
H	0.23320000	-0.71830000	-0.73260000
O	-0.39490000	0.00250000	-0.92210000
H	-0.30150000	0.61340000	-0.16790000
H	2.55090000	-0.46300000	0.43890000
O	3.18570000	0.27090000	0.36010000
H	2.64380000	1.08150000	0.31430000
O	1.29060000	2.49800000	-0.24680000
C	0.60490000	3.52610000	-0.17020000
N	0.83120000	4.45500000	0.76910000
C	-0.49890000	3.78010000	-1.17550000
H	1.53110000	4.19620000	1.45300000
C	-0.01700000	5.59500000	1.04790000
N	-0.59330000	2.65330000	-2.06290000
H	-0.28040000	4.70170000	-1.73360000
H	-1.43790000	3.93910000	-0.63720000
H	-0.99130000	5.28040000	1.43910000
H	-0.17180000	6.19250000	0.14540000
H	0.47850000	6.22180000	1.78800000
C	-1.65960000	2.49770000	-2.87440000
H	0.03490000	1.87260000	-1.89090000
C	-1.54680000	1.36010000	-3.87400000
O	-2.60010000	3.29220000	-2.88570000
H	-0.89940000	0.56270000	-3.50420000
H	-1.12880000	1.75990000	-4.80250000
H	-2.54160000	0.96320000	-4.08310000
H	-3.49080000	4.30660000	-0.06560000

C	-4.39260000	4.88710000	0.17300000
C	-5.51000000	3.92000000	0.45360000
C	-4.72530000	5.86270000	-0.95250000
H	-4.16530000	5.41240000	1.10910000
O	-5.31540000	2.97160000	1.33720000
O	-6.60080000	4.06270000	-0.08490000
H	-5.61140000	6.45110000	-0.70560000
H	-3.88840000	6.54430000	-1.12760000
H	-4.92900000	5.32010000	-1.87880000
H	-4.28610000	2.63500000	1.35040000
C	-2.97390000	1.89800000	1.43050000
N	-1.84240000	2.41740000	1.25640000
C	-2.91610000	0.57720000	2.03760000
O	-0.76040000	1.29780000	1.82960000
C	-1.53670000	0.33960000	2.25400000
C	-3.88120000	-0.37210000	2.37400000
C	-1.11280000	-0.83350000	2.88430000
H	-4.93600000	-0.21840000	2.18140000
C	-3.40540000	-1.53930000	2.96400000
C	-2.05780000	-1.77590000	3.20000000
H	-0.05570000	-1.01720000	3.05660000
N	-4.36350000	-2.58410000	3.28830000
H	-1.76700000	-2.71430000	3.65780000
O	-5.53730000	-2.40580000	2.99460000
O	-3.95580000	-3.59800000	3.83780000

1-Q50A (QM)

C	-1.98370000	-4.01590000	-2.07330000
H	-2.08860000	-3.41230000	-2.97760000
C	-0.54460000	-4.31530000	-1.71280000
C	-2.57590000	-3.28620000	-0.87350000
H	-2.50350000	-4.96580000	-2.22740000
N	0.43810000	-3.58120000	-2.26740000
O	-0.28780000	-5.12120000	-0.81370000
C	-4.03620000	-2.96730000	-0.99580000
H	-2.01800000	-2.35460000	-0.71160000
H	-2.39510000	-3.90480000	0.01280000
C	1.79210000	-3.66730000	-1.71880000
C	0.38940000	-2.75150000	-3.46120000
C	-5.08650000	-3.84640000	-0.99970000
C	-4.58060000	-1.65260000	-1.12400000
C	2.61410000	-2.79580000	-2.65070000
H	2.12690000	-4.70980000	-1.71700000
C	1.76140000	-3.11340000	-0.30790000
C	1.83170000	-2.83580000	-3.92430000
H	0.10270000	-1.72170000	-3.21310000
H	-0.31500000	-3.15070000	-4.19220000
N	-6.26430000	-3.14570000	-1.08480000
H	-5.09030000	-4.92480000	-0.92130000
C	-5.97890000	-1.80620000	-1.14710000
C	-4.02400000	-0.36830000	-1.19560000
H	2.64610000	-1.77550000	-2.25160000
H	3.63970000	-3.15820000	-2.74770000
O	1.45360000	-1.94930000	-0.06340000
N	2.11300000	-3.99520000	0.65730000
H	2.09410000	-2.03290000	-4.61760000
H	1.98570000	-3.79000000	-4.44010000
H	-7.18890000	-3.54980000	-1.08970000
C	-6.84050000	-0.71640000	-1.23240000
H	-2.94610000	-0.22180000	-1.16700000
C	-4.88350000	0.69960000	-1.27580000
C	1.97070000	-3.64330000	2.05660000
H	2.08510000	-4.97620000	0.40980000
H	-7.91830000	-0.84980000	-1.24400000
C	-6.26600000	0.51800000	-1.30180000
H	-4.49540000	1.71440000	-1.32550000
C	2.87280000	-2.46920000	2.42780000
H	0.94470000	-3.34040000	2.28650000
H	2.23330000	-4.51390000	2.66090000
H	-6.88440000	1.41040000	-1.33030000
O	2.43610000	-1.49230000	3.01820000
N	4.17770000	-2.61630000	2.07880000
C	5.16290000	-1.61230000	2.41090000
H	4.48060000	-3.53420000	1.77670000
C	5.87220000	-1.00080000	1.20780000
H	4.65590000	-0.80870000	2.94400000
H	5.94260000	-2.02500000	3.06020000
O	6.49000000	0.05610000	1.34510000
N	5.91310000	-1.69470000	0.05060000
C	6.84400000	-1.30590000	-0.99590000
H	5.36070000	-2.53380000	-0.05800000
C	6.44510000	-0.07220000	-1.79860000
H	7.81570000	-1.07450000	-0.55270000
H	6.96740000	-2.14150000	-1.68650000
O	7.25720000	0.36870000	-2.60890000
N	5.24490000	0.49800000	-1.56030000
C	4.91020000	1.81940000	-2.06910000
H	4.60170000	0.11320000	-0.86200000
C	4.91060000	2.90540000	-1.00240000
H	5.63560000	2.08620000	-2.83990000
H	3.91370000	1.81610000	-2.52010000
O	4.52110000	4.03350000	-1.30900000
N	5.33350000	2.58560000	0.22580000
H	5.68320000	1.65440000	0.44400000
C	5.31530000	3.60570000	1.25290000
H	4.37370000	4.15870000	1.15780000
C	5.43850000	3.00540000	2.64900000
H	6.12280000	4.32830000	1.07810000
H	6.40000000	2.48120000	2.72000000
C	4.31870000	2.04550000	2.99760000
H	5.47660000	3.83100000	3.36770000
H	4.23220000	1.24900000	2.25340000
H	4.48430000	1.58780000	3.97830000
H	3.35380000	2.56610000	3.03280000
H	2.53580000	-0.48620000	0.36140000
O	3.18820000	0.23830000	0.34260000
H	2.66250000	1.05840000	0.28860000
O	1.30110000	2.50620000	-0.29680000
C	0.60260000	3.52310000	-0.20280000
N	0.82370000	4.44940000	0.73940000
C	-0.50030000	3.77550000	-1.20910000
H	1.53650000	4.20140000	1.41370000
C	-0.01540000	5.59720000	1.00780000
N	-0.59000000	2.66040000	-2.10520000
H	-0.29450000	4.71020000	-1.75070000
H	-1.44770000	3.91420000	-0.67880000
H	-0.99420000	5.29420000	1.39710000
H	-0.16100000	6.19280000	0.10240000
H	0.48160000	6.22280000	1.74790000
C	-1.66340000	2.48940000	-2.90380000
H	0.19500000	2.02140000	-2.10970000
C	-1.55260000	1.34820000	-3.89940000
O	-2.64270000	3.23020000	-2.85800000
H	-0.63900000	0.76450000	-3.77830000
H	-1.57720000	1.76880000	-4.90780000
H	-2.42140000	0.69420000	-3.78780000
H	-3.51050000	4.29960000	-0.14870000
C	-4.39230000	4.89510000	0.13550000
C	-5.51260000	3.93140000	0.41750000
C	-4.72280000	5.86690000	-0.99460000

H	-4.12210000	5.41990000	1.05810000
O	-5.51190000	3.38040000	1.55530000
O	-6.33980000	3.72130000	-0.50320000
H	-5.59850000	6.47230000	-0.74070000
H	-3.88780000	6.54500000	-1.20100000
H	-4.95820000	5.31260000	-1.90410000
H	-3.78960000	2.53960000	1.24370000
C	-2.92430000	1.93000000	1.49390000
N	-1.71740000	2.39670000	1.41540000
C	-2.93200000	0.60540000	2.05080000
O	-0.84730000	1.41340000	1.90910000
C	-1.58290000	0.35950000	2.29410000
C	-3.89110000	-0.35700000	2.35520000
C	-1.12370000	-0.82320000	2.86660000
H	-4.94570000	-0.21260000	2.15380000
C	-3.41840000	-1.52380000	2.94820000
C	-2.07130000	-1.76180000	3.18530000
H	-0.06410000	-1.00070000	3.03070000
N	-4.38410000	-2.57470000	3.26890000
H	-1.77810000	-2.70530000	3.63040000
O	-5.54960000	-2.39670000	2.95670000
O	-3.97410000	-3.57680000	3.83050000

1-Q50A TS (QM)

C	-1.96540000	-4.02910000	-2.06960000
H	-2.07040000	-3.42820000	-2.97560000
C	-0.52600000	-4.32350000	-1.70610000
C	-2.56150000	-3.29770000	-0.87270000
H	-2.48230000	-4.98080000	-2.22200000
N	0.45540000	-3.58810000	-2.26120000
O	-0.26830000	-5.12660000	-0.80470000
C	-4.02240000	-2.98320000	-0.99810000
H	-2.00650000	-2.36420000	-0.71180000
H	-2.38080000	-3.91340000	0.01570000
C	1.80870000	-3.66890000	-1.71040000
C	0.40640000	-2.76200000	-3.45740000
C	-5.07030000	-3.86520000	-1.00120000
C	-4.57020000	-1.67040000	-1.13070000
C	2.62990000	-2.79780000	-2.64340000
H	2.14630000	-4.71050000	-1.70540000
C	1.77460000	-3.11150000	-0.30100000
C	1.84960000	-2.84350000	-3.91800000
H	0.11610000	-1.73240000	-3.21270000
H	-0.29570000	-3.16540000	-4.18840000
N	-6.25000000	-3.16800000	-1.09020000
H	-5.07130000	-4.94340000	-0.91990000
C	-5.96810000	-1.82790000	-1.15550000
C	-4.01710000	-0.38480000	-1.20490000
H	2.65840000	-1.77630000	-2.24690000
H	3.65660000	-3.15770000	-2.73770000
O	1.46490000	-1.94740000	-0.06020000
N	2.12750000	-3.99010000	0.66720000
H	2.11080000	-2.04180000	-4.61320000
H	2.00700000	-3.79870000	-4.43090000
H	-7.17350000	-3.57480000	-1.09480000
C	-6.83250000	-0.74070000	-1.24500000
H	-2.94070000	-0.23310000	-1.17020000
C	-4.87940000	0.68050000	-1.28930000
C	1.98170000	-3.63420000	2.06520000
H	2.09880000	-4.97180000	0.42260000
H	-7.90990000	-0.87620000	-1.26000000
C	-6.26140000	0.49500000	-1.31700000
H	-4.47790000	1.68940000	-1.34890000
C	2.88000000	-2.45660000	2.43470000
H	0.95450000	-3.33200000	2.29220000
H	2.24540000	-4.50210000	2.67280000
H	-6.89590000	1.37620000	-1.36690000
O	2.44320000	-1.48060000	3.02540000
N	4.18600000	-2.60090000	2.08610000
C	5.16760000	-1.59350000	2.41880000
H	4.49190000	-3.51830000	1.78580000
C	5.87710000	-0.98330000	1.21520000
H	4.65730000	-0.79020000	2.94910000
H	5.94750000	-2.00240000	3.07040000
O	6.49230000	0.07540000	1.35040000
N	5.92150000	-1.68010000	0.05980000
C	6.85320000	-1.29160000	-0.98620000
H	5.37020000	-2.52010000	-0.04780000
C	6.45200000	-0.06130000	-1.79300000
H	7.82330000	-1.05610000	-0.54190000
H	6.98030000	-2.12890000	-1.67410000
O	7.26410000	0.37950000	-2.60330000
N	5.24990000	0.50610000	-1.55800000
C	4.91240000	1.82540000	-2.07080000
H	4.60590000	0.12110000	-0.86060000
C	4.90800000	2.91420000	-1.00720000
H	5.63840000	2.09200000	-2.84110000
H	3.91660000	1.81800000	-2.52340000
O	4.51720000	4.04080000	-1.31780000
N	5.33030000	2.59910000	0.22250000
H	5.67950000	1.66860000	0.44440000
C	5.30740000	3.62170000	1.24680000
H	4.36430000	4.17160000	1.14880000
C	5.43020000	3.02560000	2.64470000
H	6.11310000	4.34630000	1.07130000
H	6.39300000	2.50420000	2.71830000
C	4.31250000	2.06350000	2.99430000
H	5.46520000	3.85320000	3.36130000
H	4.22670000	1.26660000	2.25050000
H	4.47940000	1.60680000	3.97510000
H	3.34660000	2.58230000	3.02970000
H	2.53310000	-0.46970000	0.36890000
O	3.18410000	0.25550000	0.33760000
H	2.65640000	1.07440000	0.28040000
O	1.31040000	2.51630000	-0.31910000
C	0.59700000	3.52200000	-0.21570000
N	0.80690000	4.44500000	0.73160000
C	-0.50460000	3.76910000	-1.22460000
H	1.52220000	4.20220000	1.40480000
C	-0.02840000	5.59790000	0.98820000
N	-0.57440000	2.67130000	-2.14340000
H	-0.31630000	4.71820000	-1.74680000
H	-1.45460000	3.87520000	-0.69160000
H	-1.02230000	5.29620000	1.33790000
H	-0.13610000	6.21050000	0.08860000
H	0.44820000	6.20510000	1.75650000
C	-1.66190000	2.47460000	-2.91720000
H	0.21660000	2.03970000	-2.15290000
C	-1.54630000	1.33120000	-3.90970000
O	-2.65860000	3.19070000	-2.84720000
H	-0.59750000	0.79800000	-3.83560000
H	-1.65140000	1.73780000	-4.91860000
H	-2.37130000	0.63310000	-3.74310000
H	-3.50340000	4.29500000	-0.12580000
C	-4.40210000	4.88120000	0.11100000
C	-5.51980000	3.91570000	0.39480000
C	-4.73350000	5.84900000	-1.02210000

H	-4.17120000	5.41090000	1.04330000
O	-5.35980000	3.02700000	1.34860000
O	-6.58160000	4.00040000	-0.20820000
H	-5.62140000	6.43790000	-0.78190000
H	-3.89720000	6.53110000	-1.19790000
H	-4.93280000	5.30010000	-1.94530000
H	-4.33930000	2.70480000	1.42220000
C	-3.00800000	1.96120000	1.52860000
N	-1.89690000	2.52960000	1.42210000
C	-2.93400000	0.61020000	2.06080000
O	-0.78500000	1.38450000	1.97020000
C	-1.54850000	0.39140000	2.29890000
C	-3.88980000	-0.36340000	2.34630000
C	-1.12180000	-0.82040000	2.86280000
H	-4.94310000	-0.21830000	2.13920000
C	-3.41460000	-1.52730000	2.94280000
C	-2.06720000	-1.76100000	3.18270000
H	-0.06450000	-1.00560000	3.03120000
N	-4.36830000	-2.57740000	3.25190000
H	-1.77400000	-2.70600000	3.62530000
O	-5.54040000	-2.40860000	2.94240000
O	-3.96280000	-3.58880000	3.80930000

Python script for ΔΔG predictions with PyRosetta

```
def mutate_repack_func4(pose, target_position, mutant, repack_radius, sfxn, ddg_bbnbrs=1,
verbose=False, cartesian=True, max_iter=None):
    import time
    from pyrosetta.rosetta.core.pack.task import operation

    #logger.warning("Interface mode not implemented (should be added!)")

    if cartesian:

sfxn.set_weight(pyrosetta.rosetta.core.scoring.ScoreTypeManager.score_type_from_name('cart_bon
ded'), 0.5)
        #sfxn.set_weight(atom_pair_constraint, 1)#0.5

sfxn.set_weight(pyrosetta.rosetta.core.scoring.ScoreTypeManager.score_type_from_name('pro_clos
e'), 0)

#logger.warning(pyrosetta.rosetta.basic.options.get_boolean_option('ex1'))#set_boolean_option(
'-ex1', True )
        #pyrosetta.rosetta.basic.options.set_boolean_option( 'ex2', True )

    #Cloning of the pose including all settings
    working_pose = pose.clone()

    #Select mutant residue
    mutant_selector                                                                          =
pyrosetta.rosetta.core.select.residue_selector.ResidueIndexSelector(target_position)

    #Select all except mutant
    all_nand_mutant_selector                                                                 =
pyrosetta.rosetta.core.select.residue_selector.NotResidueSelector()
    all_nand_mutant_selector.set_residue_selector(mutant_selector)

    #Select neighbors with mutant
    nbr_or_mutant_selector                                                                   =
pyrosetta.rosetta.core.select.residue_selector.NeighborhoodResidueSelector()
    nbr_or_mutant_selector.set_focus(str(target_position))
    nbr_or_mutant_selector.set_distance(repack_radius)
    nbr_or_mutant_selector.set_include_focus_in_subset(True)

    #Select mutant and it's sequence neighbors
    seq_nbr_or_mutant_selector                                                               =
pyrosetta.rosetta.core.select.residue_selector.PrimarySequenceNeighborhoodSelector(ddg_bbnbrs,
ddg_bbnbrs, mutant_selector, False)

    #Select mutant, it's seq neighbors and it's surrounding neighbors
    seq_nbr_or_nbr_or_mutant_selector                                                        =
pyrosetta.rosetta.core.select.residue_selector.OrResidueSelector()
    seq_nbr_or_nbr_or_mutant_selector.add_residue_selector(seq_nbr_or_mutant_selector)
    seq_nbr_or_nbr_or_mutant_selector.add_residue_selector(nbr_or_mutant_selector)

    if verbose:
        print(f'mutant_selector:
{pyrosetta.rosetta.core.select.residue_selector.selection_positions(mutant_selector.apply(work
ing_pose))}')
```

```
        print(f'all_nand_mutant_selector:
{pyrosetta.rosetta.core.select.residue_selector.selection_positions(all_nand_mutant_selector.a
pply(working_pose))}')
        print(f'nbr_or_mutant_selector:
{pyrosetta.rosetta.core.select.residue_selector.selection_positions(nbr_or_mutant_selector.app
ly(working_pose))}')
        print(f'seq_nbr_or_mutant_selector:
{pyrosetta.rosetta.core.select.residue_selector.selection_positions(seq_nbr_or_mutant_selector
.apply(working_pose))}')
        print(f'seq_nbr_or_nbr_or_mutant_selector:
{pyrosetta.rosetta.core.select.residue_selector.selection_positions(seq_nbr_or_nbr_or_mutant_s
elector.apply(working_pose))}')


    #Mutate residue and pack rotamers before relax
    #if list(pose.sequence())[target_position-1] != mutant:
        #generate packer task
    tf = TaskFactory()
    tf.push_back(operation.InitializeFromCommandline())
    tf.push_back(operation.IncludeCurrent())

    #Set all residues except mutant to false for design and repacking
    prevent_repacking_rlt = operation.PreventRepackingRLT()
    prevent_subset_repacking     =     operation.OperateOnResidueSubset(prevent_repacking_rlt,
all_nand_mutant_selector, False )
    tf.push_back(prevent_subset_repacking)

    #Assign mutant residue to be designed and repacked
    resfile_comm                                                                             =
pyrosetta.rosetta.protocols.task_operations.ResfileCommandOperation(mutant_selector,    f"PIKAA
{mutant}")
    resfile_comm.set_command(f"PIKAA {mutant}")
    tf.push_back(resfile_comm)

    #Apply packing of rotamers of mutant
    packer = pyrosetta.rosetta.protocols.minimization_packing.PackRotamersMover()
    packer.score_function(sfxn)
    packer.task_factory(tf)
    if verbose:
        logger.warning(tf.create_task_and_apply_taskoperations(working_pose))
    packer.apply(working_pose)

    #allow the movement for bb for the mutant + seq. neighbors, and sc for neigbor in range,
seq. neighbor and mutant
    movemap = pyrosetta.rosetta.core.select.movemap.MoveMapFactory()
    movemap.all_jumps(False)
    movemap.add_bb_action(pyrosetta.rosetta.core.select.movemap.mm_enable,
seq_nbr_or_mutant_selector)
    movemap.add_chi_action(pyrosetta.rosetta.core.select.movemap.mm_enable,
seq_nbr_or_nbr_or_mutant_selector)

    #for checking if all has been selected correctly
    #if verbose:
    mm  = movemap.create_movemap_from_pose(working_pose)

    logger.info(mm)

    #Generate a TaskFactory
```

```python
    tf = TaskFactory()
    tf.push_back(operation.InitializeFromCommandline())
    tf.push_back(operation.IncludeCurrent())
    #tf.push_back(operation.NoRepackDisulfides())

    #prevent all residues except selected from design and repacking
    prevent_repacking_rlt = operation.PreventRepackingRLT()
    prevent_subset_repacking     =     operation.OperateOnResidueSubset(prevent_repacking_rlt,
seq_nbr_or_nbr_or_mutant_selector, True )
    tf.push_back(prevent_subset_repacking)

    # allow selected residues only repacking (=switch off design)
    restrict_repacking_rlt = operation.RestrictToRepackingRLT()
    restrict_subset_repacking   =   operation.OperateOnResidueSubset(restrict_repacking_rlt   ,
seq_nbr_or_nbr_or_mutant_selector, False)
    tf.push_back(restrict_subset_repacking)


    #Perform a FastRelax
    fastrelax = pyrosetta.rosetta.protocols.relax.FastRelax()
    fastrelax.set_scorefxn(sfxn)

    if cartesian:
        fastrelax.cartesian(True)
    if max_iter:
        fastrelax.max_iter(max_iter)

    fastrelax.set_task_factory(tf)
    fastrelax.set_movemap_factory(movemap)
    fastrelax.set_movemap_disables_packing_of_fixed_chi_positions(True)

    if verbose:
        logger.info(tf.create_task_and_apply_taskoperations(working_pose))
    fastrelax.apply(working_pose)
    return working_pose

def cart_ddg(site,res):

    newpose = pose.clone()
    scores = []

    for i in range(3): #ONLY RUNS ONCE!!!!
        scorefxn = create_score_function("ref2015_cart")
        newpose = mutate_repack_func4(newpose,site, res, 6, scorefxn,verbose = False, cartesian
= True)
        news = scorefxn(newpose)
        scores.append(news)
    return scores

all_as = sorted(list(set(pose.sequence())))
sites = np.arange(1,len(pose.sequence()) + 1)

assert len(all_as) == 20

inputs_ = [[site,res] for site in sites for res in all_as]
print(len(inputs_))
cores = 60 # os.cpu_count()
print(cores)
```

```
with multiprocessing.Pool(processes=cores) as pool:
    results = pool.starmap(cart_ddg,inputs_)

import pickle
with open('hg34_base.pkl', 'wb') as f:
    pickle.dump(results, f)
```

SI References

1. Otten, R. *et al.* How directed evolution reshapes the energy landscape in an enzyme to boost catalysis. *Science* **370**, 1442–1446 (2020).
